# An Extremely Stable Interprotein Tetrahedral Hg(Cys)_4_ Core Forms in the Zinc Hook Domain of Rad50 Protein at Physiological pH

**DOI:** 10.1002/chem.202202738

**Published:** 2022-11-07

**Authors:** Marek Łuczkowski, Michał Padjasek, Józef Ba Tran, Lars Hemmingsen, Olga Kerber, Jelena Habjanič, Eva Freisinger, Artur Krężel

**Affiliations:** ^1^ Department of Chemical Biology Faculty of Biotechnology University of Wrocław Joliot-Curie 14a 50-383 Wrocław Poland; ^2^ Department of Chemistry University of Copenhagen Universitetsparken 5 2100 København Ø Denmark; ^3^ Department of Chemistry University of Zurich Winterthurerstrasse 190 8057 Zürich Switzerland

**Keywords:** affinity, Cys-rich protein, mercury toxicity, metal-sulfur cluster, stability constant

## Abstract

In nature, thiolate‐based systems are the primary targets of divalent mercury (Hg^II^) toxicity. The formation of Hg(Cys)_*x*_ cores in catalytic and structural protein centers mediates mercury's toxic effects and ultimately leads to cellular damage. Multiple studies have revealed distinct Hg^II^‐thiolate coordination preferences, among which linear Hg^II^ complexes are the most commonly observed in solution at physiological pH. Trigonal or tetrahedral geometries are formed at basic pH or in tight intraprotein Cys‐rich metal sites. So far, no interprotein tetrahedral Hg^II^ complex formed at neutral pH has been reported. Rad50 protein is a part of the multiprotein MRN complex, a major player in DNA damage‐repair processes. Its central region consists of a conserved CXXC motif that enables dimerization of two Rad50 molecules by coordinating Zn^II^. Dimerized motifs form a unique interprotein zinc hook domain (Hk) that is critical for the biological activity of the MRN. Using a series of length‐differentiated peptide models of the *Pyrococcus furiosus* zinc hook domain, we investigated its interaction with Hg^II^. Using UV‐Vis, CD, PAC, and ^199^Hg NMR spectroscopies as well as anisotropy decay, we discovered that all Rad50 fragments preferentially form homodimeric Hg(Hk)_2_ species with a distorted tetrahedral HgS_4_ coordination environment at physiological pH; this is the first example of an interprotein mercury site displaying tetrahedral geometry in solution. At higher Hg^II^ content, monomeric HgHk complexes with linear geometry are formed. The Hg(Cys)_4_ core of Rad50 is extremely stable and does not compete with cyanides, NAC, or DTT. Applying ITC, we found that the stability constant of the Rad50 Hg(Hk)_2_ complex is approximately three orders of magnitude higher than those of the strongest Hg^II^ complexes known to date.

## Graphical abstract

**Hg^II^ toxicity**: Hg^II^ targets the intermolecular Zn^II^‐binding site of DNA binding protein Rad50 to form the strongest metal complex in the living world and jeopardize the DNA repair mechanism.

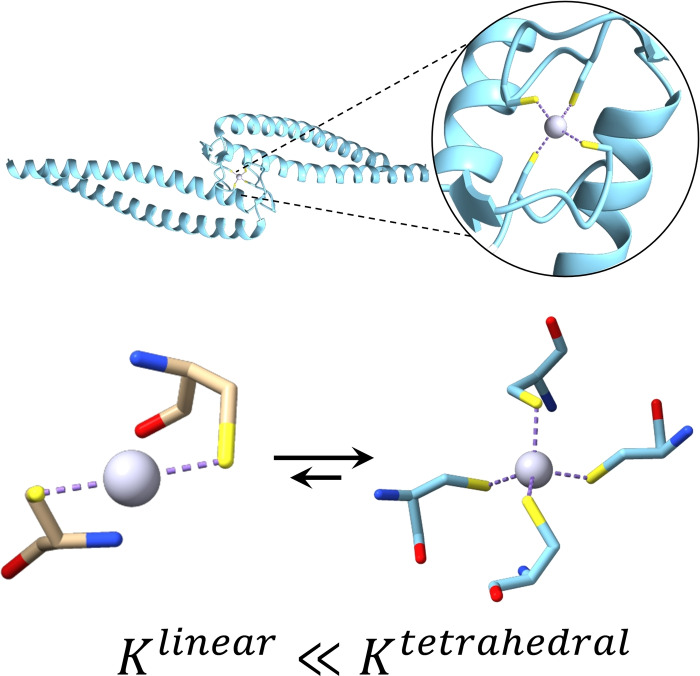

## Introduction

Divalent mercury (Hg^II^) does not have strict preferences with respect to coordination number, although the most typical are 2, 3, or 4 depending on the type and spatial orientation of the ligands.^[1]^ During complexation in an aqueous environment Hg^II^ tends to achieve linear geometry as it allows for formation of the shortest (strongest) bond,[^2^, ^3^, ^4^] yielding the highest energy stabilization.^[5]^ Digonal coordination tends to keep the optimal angle between the bonds close to 180°. The increase in coordination number with ligands utilizing the same binding atom coincides with an increase of the bond length. In practice, three‐ and four‐coordinate Hg^II^ species are always distorted, regardless of the symmetry enforcement of the binding moiety. Furthermore, the discrepancy of the bond lengths is compensated, as the increase of one Hg−S bond length is counterbalanced by shortening of another one.^[6]^ The most extreme example of the distortion is the T‐shaped geometry of a three‐coordinate species with two short Hg−S bonds and one weaker and longer oriented perpendicularly.^[7]^ The bond lengths may vary significantly from the common values found for homoleptic mononuclear species when the coordinating atom bridges Hg^II^ in a dimer or repetitive dimeric center of the polymer.

In nature, thiolate‐based systems are the most common targets for Hg^II^.^[8]^ No biological functions of mercury have been found so far, and exposure to this metal leads to mercury poisoning.^[9]^ Therefore, a binding event is usually related to toxic actions. On the other hand, thiolates are also engaged in metal sequestration and bioremediation mechanisms, transmembrane transport in bacteria (Mer protein superfamily)^[10]^ and intracellular metal scavenging systems (e. g., metallothioneins)[^11^, ^12^] in eukaryotic cells. At neutral pH conditions the low molecular weight compounds with one (*N*‐acetylcysteine)^[13]^ or two (dithiothreitol) thiol groups form digonal Hg^II^ complexes as metal enforces its preferred binding mode. This phenomenon occurs even at higher ligand‐to‐metal ratios, where the HgS_2_ coordination environment, though in a mixture with HgS_3_, is still the predominant species in the solution.^[14]^ Nevertheless, when the thiol ligands are structurally associated within a single polypeptide chain, where they are in a predefined spatial orientation and may sequester metal ions even in their protonated state,^[2]^ the Hg^II^ binding mode is determined by the ligand. Mercury‐substituted rubredoxin, which sequesters the metal ion within a homoleptic tetrahedral thiolate HgS_4_ environment,^[15]^ or metallothioneins forming metallic clusters engaging HgS_2–4_ binding modes are good examples of this phenomenon.[^16^, ^17^, ^18^] It is worth noting that these are the only examples of intramolecular Hg(Cys)_4_ protein centers existing in the neutral pH range. Although the soft thiolates are the major ligand donor atoms for Hg^II^ coordination, the toxic action of the metal may also affect mixed‐ligand[^19^, ^20^] or histidine‐based enzymatic sites.^[21]^

Intermolecular Hg^II^ binding to macromolecules is usually related to the toxic activity of the metal and mechanistically relies on the formation of aggregates. Also here, in most cases the metal is majorly bound to thiolate groups,[^22^, ^23^, ^24^] though the metal promoted aggregation may as well be fulfilled through binding to other medium/soft donors such as imidazole nitrogen.^[25]^ Formation of digonal Hg^II^ species is observed regardless of the accessibility of potential binding sites. Species of higher coordination number are formed at non‐physiological pH conditions (pH>9.0) that enforce proton dissociation from the adjacent residues and their subsequent binding to the metal center.^[26]^ The relationship between Hg^II^ binding preferences of the protein and pH conditions is particularly manifested in solution, where the Hg^II^ sequestration pattern at the protein interface may differ from the solid state structure. Solid state studies of mercurated HAH1, a human copper chaperone, performed for species in the neutral pH range, suggest the presence of distorted trigonal or tetrahedral Hg^II^ sites formed within the protein dimer,^[27]^ while solution studies reveal the formation of linear Hg^II^ complexes in similar pH conditions.^[28]^

**Figure 1:**
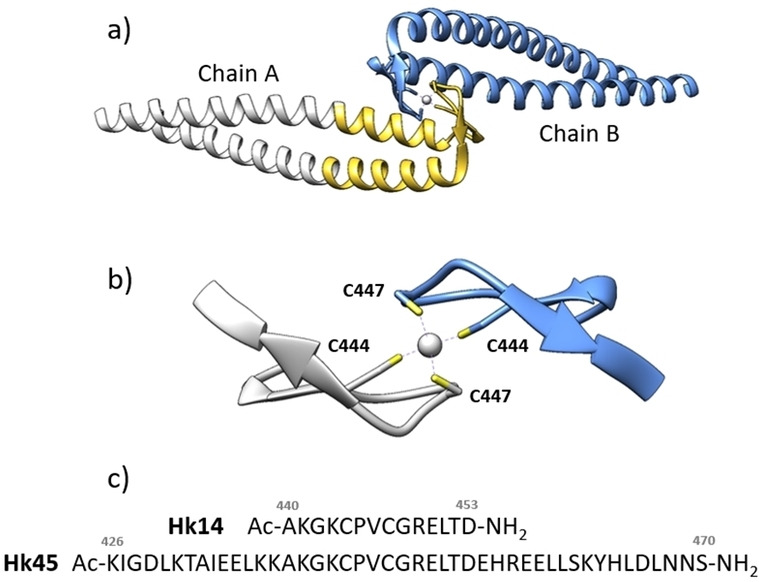
Structural representation of the hook domain from *P. furiosus* Rad50 and protein fragments used in this study. a) Crystal structure representation of the Hg^II^ complex with a 112 amino acid fragment (PDF ID: 1 L8D).^[32]^ The yellow color in chain A highlights the Hk45 fragment. b) Central 14‐aa fragment corresponding in length to Hk14. c) Sequences of Hk14 and Hk45 model peptides. The amino acid sequence of Hk130 is given in the Supporting Information.

Interprotein metal binding sites are formed by at least two protein molecules (or entities?) interacting in homo‐or heteromeric mode. The binding of a metal ion at protein interfaces is frequently related to distinctly different properties that either provide a critical scaffold for organization of a heteromeric multi‐subunit system or trigger a molecular switch.[^29^, ^30^] Of note, within the assortment of natural interprotein metal binding sites, those incorporating Zn^II^ are the most common.^[24]^ Among those, the highly conserved zinc hook domain of the Rad50 protein has undergone the most comprehensive characterization of biological, structural and physicochemical properties (Figure 1a).[^31^, ^32^, ^33^] It is a component of the evolutionary conserved MR(N/X) (Mre11, Rad50, Xrs2/Nbs1) complex that plays a pivotal role in double‐stranded DNA damage signaling and repair (Figure S1 in the Supporting Information).[^34^, ^35^, ^36^, ^37^]

It consists of a long antiparallel coiled‐coil protruding from the globular ATPase/DNA‐binding domain that ends with a small β‐hairpin loop containing a Cys‐Xaa‐Xaa‐Cys (CXXC) motif involved in Zn^II^ coordination (Figure 1b).

Two such motifs from separate Rad50 molecules form a unique Zn(Cys)_4_ interprotein core. This small interface together with surrounding electrostatic and hydrophobic interactions forms the zinc hook domain (Zn(Hk)_2_) that displays remarkably high complex stability due to the Zn^II^‐coupled folding process.[^38^, ^39^, ^40^, ^41^] Solid state characterization of the central fragment of the Rad50 zinc hook domain from *Pyrococcus furiosus* took advantage of Hg^II^ as a probe for Zn^II^.^[32]^ The four cysteine thiolates, two from each monomer coordinate the Hg^II^ ion in a distorted tetrahedral coordination sphere and increased the resolution of the crystal structure. However, as previous studies on similar systems with Zn^II^ and Cd^II^ suggest,^[28]^ this phenomenon does not necessarily occur in solution and dynamic equilibria occur.[^33^, ^39^] To clarify this issue, in this study we performed state‐of‐the‐art studies on Hg^II^ sequestration using functional Rad50 hook domain models that have been successfully applied before to study sequence‐structure‐stability relations in this zinc domain.[^33^, ^39^] We provide solid evidence that Rad50 is unique in its Hg^II^ binding properties, when compared to other similar systems, and preferably sequesters Hg^II^ within a pseudotetrahedral environment at pH conditions that would otherwise favor formation of linear species. Furthermore, against all odds, the extremely high thermodynamic stability of the Hg(Cys)_4_ complex exceeds the energy gain of the HgS_2_ coordination environment in digonal complex formation, typically the most stable form of Hg^II^ sequestration. This study also sheds a new light on the molecular basis of mercury genotoxicity,^[42]^ indicating that not only intra‐ but also interprotein zinc sites are affected by Hg^II^.

## Results and Discussion

Our previous studies on the central fragments of the Rad50 protein from *P. furiosus* revealed unique sequence‐structure‐stability relations. They indicated that a 14 amino acid long central fragment (Hk14) is sufficient to form a highly structured minimal zinc hook domain and thus constitutes a good model for the β‐hairpin formed upon Zn^II^ coordination.^[38]^ The full zinc hook domain is composed of a 45 amino acid residues long fragment (Hk45, Figure 1c) and includes all hydrophobic and electrostatic interactions.[^33^, ^38^] Finally, the fragment spanning 130 amino residues (Hk130) structurally overlaps in the initial X‐ray structures with Zn^II^ and Hg^II^.^[32]^ Therefore, here we selected these three Rad50 fragments (Hk14, Hk45, and Hk130) to probe the Hg^II^ coordination environment regarding hook model length and thermodynamic stability. Table S1 presents all Rad50 fragments, indicating those that have been N‐ or C‐terminally modified (acetylation, FAM modification or amidation).

### ^199^Hg NMR spectroscopy

**Figure 2:**
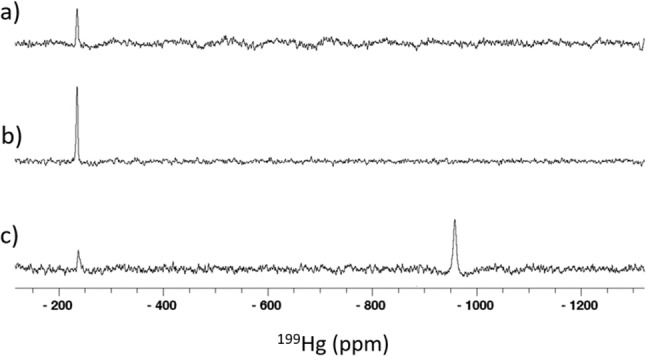
^199^Hg NMR spectra of Hg^II^ complexes of 3 mM Hk14 with Hg^II^ prepared in 10 % D_2_O/90 % H_2_O: a) Hg^II^/Hk14=1 : 2, pH 7.4; b) Hg^II^/Hk14 1 : 2, pH 8.5; c) Hg^II^/Hk14=1 : 1, pH 7.4. All spectra were recorded at 37 °C; the pH was manually adjusted to the desired values.

^199^Hg NMR measurements were used to probe the geometry of the Hg^II^ complexes of all zinc hook peptides. Even though all Hk45 samples were soluble in presence of Hg^II^, no signal were detected at any Hg^II^‐to‐peptide ratio. The reason might be the presence of intermediate exchange processes in solution that lie within the NMR timescale, leading to substantial line broadening and hence no detectable signal after spectra processing. Among the three examined Rad50 fragments, only the shortest, Hk14, gave sharp and easily detectable signals at various Hg^II^‐to‐ligand ratios and under physiological and slightly alkaline pH conditions (Figure 2).

Spectra of the sample prepared with a 2 : 1 ratio of peptide to Hg^II^ at physiological pH yield a single Hg^II^ species with a ^199^Hg shift of −236 ppm (Figure 2a) that rationalizes the presence of a distorted tetrahedral HgS_4_ core.^[20]^ Alkaline pH has a negligible effect on the chemical shift value indicating the same coordination environment of the Hg^II^ ion; however, the signal‐to‐noise ratio is slightly better (Figure 2b). A higher Hg^II^‐to‐peptide ratio of 1 : 1 leads to the formation of a new major ^199^Hg signal with a chemical shift value at −959 ppm, which agrees with the chemical shift values reported for two‐coordinate Hg^II^ species.[^43^, ^44^] In addition, for a minor, now rather broad signal characteristic for a HgS_4_ species is still observed at −239 ppm (Figure 2c).These results suggest a metal‐driven mechanism of peptide dimer formation at a metal‐to‐peptide ratio of 1 : 2, which is further stabilized by the rearrangement of the peptide scaffold, as discussed elsewhere.[^33^, ^38^, ^39^] The addition of subsequent aliquots of Hg^II^ leads to the dissociation of the complex, allowing the formation of linear HgS_2_ species that are preferably formed by Hg^II^ in the physiological pH range.^[1]^ However, one may still note the presence of a Hg(Hk14)_2_ signal, which may indicate either a small excess of peptide over the metal ion or/and the rather uncommon preference of the hook domain model for tetrahedral Hg^II^ sequestration.

### Probing HgS_*x*_ sites with ^199m^Hg PAC spectroscopy

**Figure 3:**
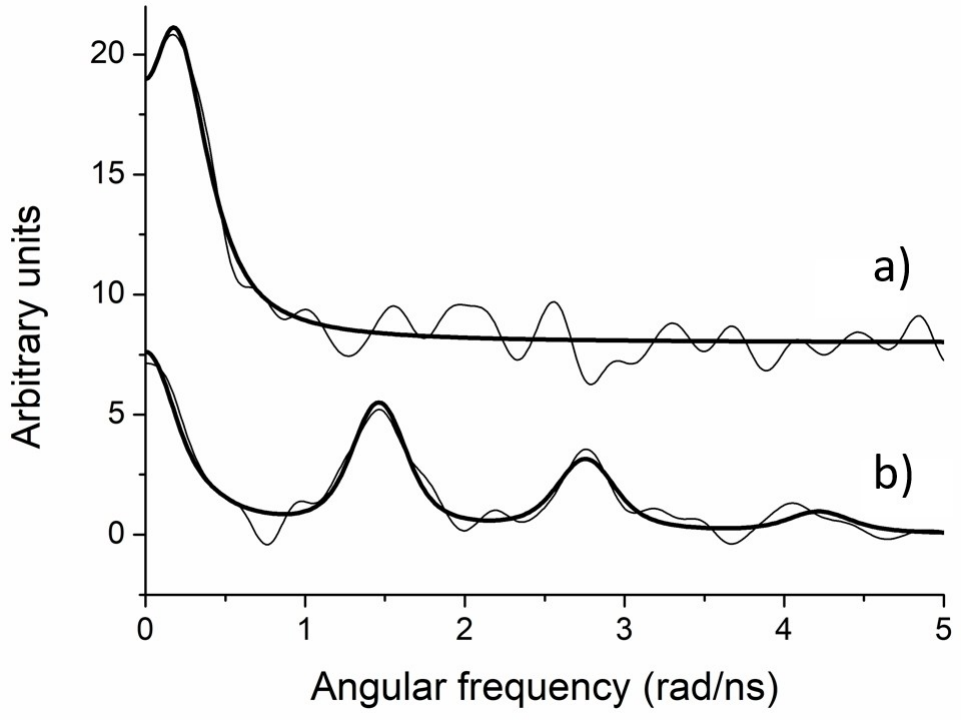
^199m^Hg PAC data for Hg^II^ complexes of Hk14 peptide; a) Hg^II^/Hk14=1 : 2, pH 8.5; b) Hg^II^/Hk14=1 : 1, pH 7.4. Fourier‐transformed experimental data and fits are shown as thin and bold lines, respectively. For more details, see Table S2.

All PAC spectra and fitted parameters are presented in Table S3 and Figures S2 and S3. In all cases, the PAC data are fitted satisfactorily with one or two nuclear quadrupole interactions (NQIs), a low frequency signal, NQI_1_, and a high frequency signal, NQI_2_. The low‐frequency signal, NQI_1_, with *ν_Q_* **=**0.1–0.2 GHz (and asymmetry parameter, *η*, fixed to 1), is in good agreement with literature data for distorted tetrahedral HgS_4_ coordination.[^15^, ^45^] The difference in the reported asymmetry parameter, *η*, is insignificant, because *η* is very difficult to determine accurately for low frequency ^199m^Hg PAC signals, simply because only one (or less than one) oscillation of the perturbation function is measurable. The high frequency signal, NQI_2_, gives a frequency (and asymmetry parameter) very similar to linear HgS_2_ for the HK14 peptide under conditions of 1 : 1 Hg(II):HK14, *ν_Q_* **=**1.48(2) GHz (*η* **=**0.22(2)), and a slightly higher frequency, *ν_Q_* **=**1.60–1.67 GHz (*η* **=**0.0–0.2) in the remaining cases.[^45^, ^47^] The higher frequency might be caused by slightly shorter Hg−S bond lengths in a HgS_2_ structure, or the signal could reflect a different species, possibly T‐shaped HgS_3_, but both interpretations are speculative, and no conclusions in this work rely on this signal. Several individual spectra were recorded for Hk14 at pH 8.5, and they displayed a systematic change from NQI_1_ (HgS_4_) towards NQI_2_ (*ν_Q_* **=**1.60–1.67 GHz) as a function of time between preparation of the peptide stock solution to the initiation of the PAC experiment, thus indicating oxidation of the cysteine thiols. Moreover, the same high frequency signal is observed in all experiments with HK45 and HK130, implying that these samples may also be partially oxidised. Consequently, only the two spectra indicating no or very little oxidation were included in the main text (Figure 3).

These data demonstrate that Hg^II^ binding to the Hk14 peptide at pH 8.5 and at a Hg^II^‐to‐peptide ratio of 1 : 2, gives a very low frequency signal, in agreement with reference data for HgS_4_ coordination.[^15^, ^45^] The low frequency component is also in accord with the distorted tetrahedral HgS_4_ geometry presented in the crystal structure of the Rad50 zinc hook.^[48]^ Contrary to this, Hg^II^ bound to the Hk14 peptide at pH 7.4 and at Hg^II^‐to‐peptide of 1 : 1 gives a high frequency signal, reflecting HgS_2_ coordination,^[46]^ possibly with a minor component (∼10 % of the total amplitude) reflecting HgS_4_ coordination, see Table S3. Thus, these spectra are in excellent agreement with the ^199^Hg NMR spectra, as well as with the UV‐Vis absorption spectra recorded in this work, see Figures 2 and 6, respectively. The remaining data series are not completely void of information despite the partial oxidation, and indicate that the Hg^II^ coordination by the Hk45 and Hk130 peptides is also HgS_4_ under condition of Hg^II^‐to‐peptide of 1 : 2 (Figure S3; although also a high‐frequency component is present). Moreover, the observed frequency of the low frequency signal is slightly higher than for the Hk14 peptide, implying that the tetrahedral HgS_4_ geometry is slightly more distorted in these longer model systems of the zinc hook (Table S3 and Figure S3).

**Figure 4:**
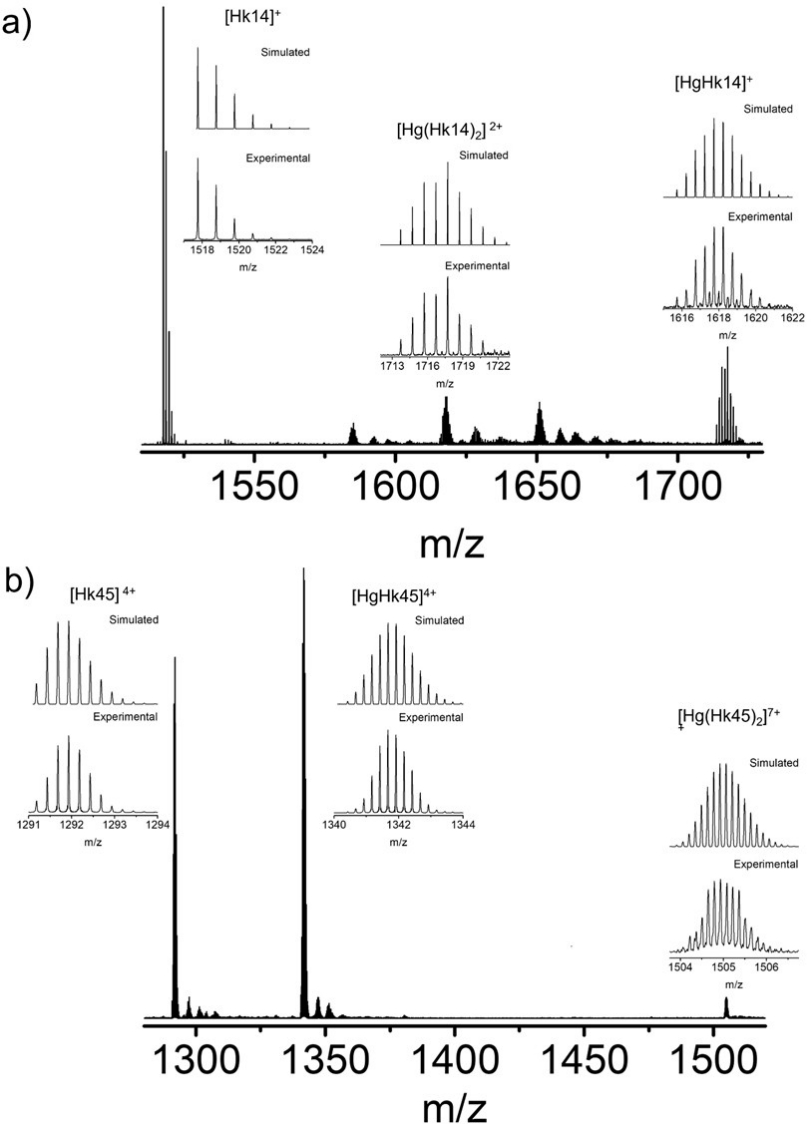
Hg^II^ complexes of Hk peptides monitored by ESI‐MS (20 mM ammonium carbonate, pH 8.0). a) [Hk14]=1×10^−4^ M; Hg^II^/Hk14 ratio 1 : 2; MeOH/H_2_O=50 : 50 and b) [Hk45]=1×10^−4^ M; Hg^II^/Hk45 ratio 1 : 2; MeOH/H_2_O=50 : 50. Insets present isotopic profiles of detected species with experimental (lower layer) and simulated (upper layer) spectra, respectively.

**Figure 5:**
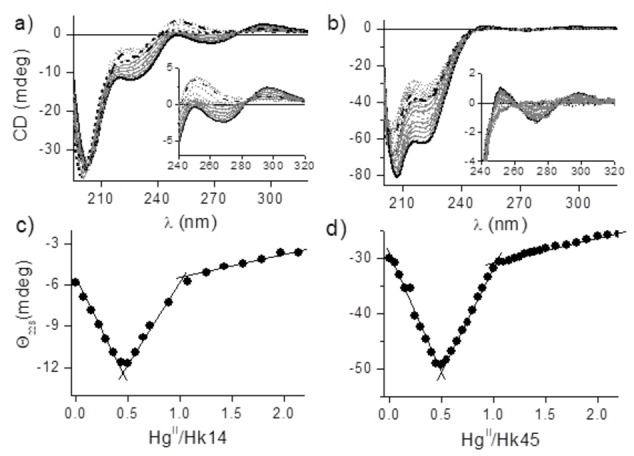
CD spectra of a) 100 μM Hk14 and b) 50 μM Hk45 Rad50 zinc hook fragments titrated with Hg^II^. Spectra were recorded in 20 mM Tris**⋅**HCl buffer, pH 7.4, 0.1 M NaClO_4_, and 150 μM TCEP. Hg(Hk)_2_ complex spectra (solid lines), HgHk complex spectra (dashed lines), oversaturated complex spectra (dotted lines), apopeptide spectra (short dashed lines). c) and d) Relation between ellipticity at 228 nm and Hg^II^/Hk molar ratios for Hk14 and Hk45, respectively.

**Figure 6:**
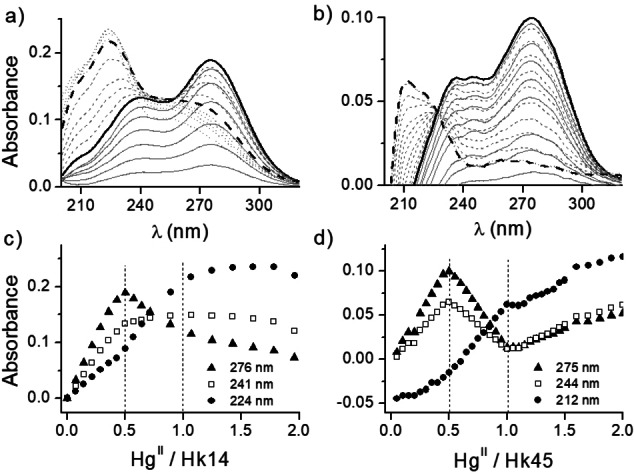
Electronic difference spectra of a) Hk14 and b) Hk45 Rad50 zinc hook fragments titrated with Hg^II^. Spectra were recorded in 20 mM Tris**⋅**HCl, pH 7.4, 0.1 M NaClO_4_, 150 μM TCEP for 100 μM Hk14 and 50 μM Hk45, respectively; Hg(Hk)_2_ complex spectra (solid lines), HgHk complex spectra (dashed lines), oversaturated complex spectra (dotted lines). Inflection points at given wavelengths which correspond to the 1 : 2 and 1 : 1 ratios of c) Hg^II^/Hk14 and d) Hg^II^/Hk45, respectively.

### MS‐monitored Hg^II^ binding to Hk peptides

Mass spectrometry‐based analysis of Hg^II^‐Hk complexation shows a mixture of mono‐ and dimeric mercurated Hk14 and Hk45 complexes at neutral pH. Electrospray ionization mass spectrometry (ESI‐MS) spectra recorded for solutions containing Hg^II^ and Hk14 in a ratio of 1 : 2 reveal signals of monomeric apo‐Hk (*m*/*z* 1517.8 Da) next to HgHk ([HgHk14]^+^; *m*/*z* 1717.7 Da) and Hg(Hk)_2_ ([Hg(Hk14)_2_]^2+^; *m*/*z* 1618.2 Da) species (Figure 4a). Samples of Hg^II^ and Hk45 mixed in the same 1 : 2 ratio yield nearly the same set of species, apo‐Hk45 ([Hk45]^4+^; *m*/*z* 1291.9 Da), HgHk45 ([HgHk45]^4+^; *m*/*z* 1341.7 Da) as well as the 1 : 2 dimer Hg(Hk45)_2_ ([Hg(Hk45)_2_]^7+^; *m*/*z* 1504.9 Da; Figure 4b).

In general, as the physical conditions of mass spectrometry measurements are rather harsh and the relatively high temperature make the thiolates more sensitive to oxidation, the equilibrium might be shifted towards the monomeric 1 : 1 complexes during the measurements.^[49]^ In consequence, the intensities of the signals for the 1 : 2 species may not represent their abundance in the sample. For that reason, the results of mass spectrometry may be interpreted only qualitatively and not quantitatively when metal speciation is examined.

### Spectropolarimetric Hg^II^ titration of Hk peptides

Far‐UV CD titrations, performed by the addition of Hg^II^ to metal‐free Hk14 and Hk45 peptides at pH 7.4, demonstrate extensive conformational changes upon Hg^II^ coordination (Figure 5). Spectra recorded up to a Hg^II^‐to‐Hk14 ratio of 1 : 2 result in intensive bands at (−) 203 and (−) 226 nm and less intensive bands (ligand‐to‐metal charged transfer, LMCT) at (−) 267 and (+) 296 nm, respectively (Figure 5a). Significant changes of CD spectra upon metal complexation and appearance of the first two bands were also observed for the Zn^II^ and Cd^II^ complexes with Hk14,[^33^, ^38^, ^39^] indicating formation short β‐hairpin in excess of Hk14 over Hg^II^. Moreover, appearance of two less energetic LMCT bands at (+) 296 and (−) 269 nm indicates the presence of distorted tetrahedral species.[^15^, ^50^, ^51^] This together confirms that Hg^II^ is tetrahedrally coordinated by Hk14, analogously to Zn^II^ and Cd^II^. Spectra of Hk45 recorded for the same range of molar ratios demonstrate an almost identical scenario: appearance of two intensive bands at (−) 207 and (−) 220 nm and less energetic bands at (−) 273 and (+) 298 nm (Figure 5b). Changes in the relative intensity of the first two negative bands demonstrate formation of an α‐helical structure. The same behavior was observed previously for Zn^II^ and Cd^II^, where the formed helices were capable of coiled‐coil structure formation. The subtraction of the apo‐ spectrum from the CD spectrum of the 1 : 2 complex of Hk45 with Hg^II^ yields a spectrum typical for coiled‐coil helices as the band intensities at 221 and 209 nm of the difference spectrum give a ratio of 1.11 (Figure S4).^[52]^

The addition of more Hg^II^ to the Hk14 sample, that is, for ratios ranging from 1 : 2 to 1 : 1 causes significant decay of all bands, especially those at 203 to 226 nm (Figure 5a). Lower energetic bands evolve to one positive at 251 nm. Since the energy of transition is surprisingly high, it is difficult to discriminate digonal from trigonal T‐shaped Hg^II^ species. By analogy, the same scenario is observed for the Hk45 fragment of the zinc hook domain; however, the negative band at 220 nm originates from the combined effect of charge transfer evolution and peptide folding, which are difficult to dissect. Regardless of the zinc hook peptide fragment used, the relations between ellipticity at 226 nm presented in Figure 5c and d clearly indicate that at least three different Hg^II^ species are formed. At low Hg^II^‐to‐peptide ratios a tetrahedral Hg(Hk)_2_ complex is formed, which turns to HgHk with digonal geometry at a ratio of 1 : 1. Additional Hg^II^ increases the CD signal decay due to a time‐dependent gradual increase of HgHk species formation or unspecific albeit still chiral binding of the excess Hg^II^ to the peptide fragment.

### Spectrophotometric Hg^II^ titrations of the Hk peptides

Hk14 and Hk45 hook peptides were titrated with Hg^II^ analogously to the CD‐monitored titrations under analog conditions (Figure 6). The observed changes in absorbance during the titration of both peptides are consistent with the CD spectra. The linear absorbance increase at 241 and 276 nm in the case of Hk14 (Figure 6a) and 243 and 275 nm for Hk45 (Figure 6b) for Hg^II^‐to‐Hk molar ratios up to 1 : 2 indicates the formation of Hg(Hk)_2_ species. The absorption maxima at these wavelengths are characteristic for spectroscopic signatures of distorted tetrahedral Hg^II^ species formed in solution under the used conditions.[^15^, ^28^] Further addition of Hg^II^ results in a decay of the aforementioned electronic transitions and evolution of new bands at 224 and 212 nm for Hk14 and Hk45, respectively, occurs, indicating formation of a digonal complex with a stoichiometry of 1 : 1 (HgHk). The decay of LMCT bands and the transition from Hg(Hk)_2_ to HgHk proceeds differently for Hk14 (Figure 6c) and Hk45 (Figure 6d) and is less linear for the former than the latter. Substantially different behavior was also observed for Zn^II^ and Cd^II^ in previous studies, probably due to the strong preference of these metal ions exclusively for tetrahedral geometry in the sulfur‐rich environment.[^33^, ^39^] The negative value of absorbance in the difference spectra in the far‐UV range indicate that Hg^II^ binding enforces some structural rearrangements of the backbone in the structured part of Hk45. Unfortunately, discrimination between intra‐ and intermolecular coordination of Hg^II^ is not possible based on the observed spectroscopic pattern. Both complex types could have crucial consequences for the biological function of the zinc hook domain of the MRN complex. Purely intramolecular coordination would lead to permanent dissociation of the functional protein dimer, while formation of a coordinatively linked dimer, structurally different from the native Zn^II^‐bound protein assembly. Therefore, the evaluation of the association forms of the peptides, both as apo and mercurated species, is essential in understanding the mechanisms of Hg^II^ toxicity related to the structure and function of the zinc hook domain of the MRN protein.

### SEC‐HPLC of Hg^II^ complexes of Hk peptides

**Figure 7:**
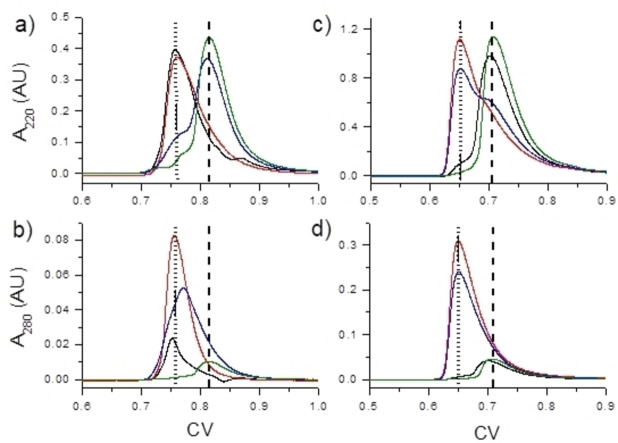
SEC‐HPLC of metal‐free and mercurated Hk14 (a and b) and Hk45 (c and d) conducted in 20 mM Tris pH 7.4 with 150 mM NaF. Prepared injected samples were: metal‐free Hk (black line), 0.45 Hg^II^/Hk molar ratio (red line), 0.9 Hg^II^/Hk molar ratio (blue line) and 1.5 Hg^II^/Hk molar ratio (green line). Chromatograms represent elution profiles as absorbance detected at wavelengths of 220 (top) and 280 nm (bottom) as a function of column volume (CV=5 mL). Dotted and dashed lines represent elution volumes corresponding to the Hg(Hk)_2_ biscomplex and HgHk monocomplex, respectively.

A size‐exclusion‐HPLC analytical approach (SEC‐HPLC) was used to evaluate the dimerization/oligomerization state of the zinc hook complexes with Hg^II^ in more detail. For this, the apo‐peptides and variously mercurated samples were prepared in 20 mM Tris buffer, pH 7.4, with 150 mM NaF, and the same buffer was used for the SEC‐HPLC analysis. The results (Figure 7) prove that both Hk14 and Hk45 peptides form an intermolecular Hg(Hk)_2_ complex at a metal‐to‐Hk molar ratio of 0.9 : 2, which start to dissociate at a ratio of 0.9 : 1 and equilibrate at 1.5 : 1 in the form of monomeric HgHk complexes.

The retention time in SEC is inversely related to the hydrodynamic radius of the substance. The 220 nm absorbance originates from the protein backbone and (Figure 7a and c) is therefore proportional to the peptide chain length. In the chromatogram, two elution peaks are observed that can be assigned to the 1 : 1 and the 1 : 2 complex, respectively (shown as dashed and dotted lines). Moreover, the absorbance at 280 nm (Figure 7b and d), is mostly affected by the Hg−S LMCT bands of the tetrathiolate HgS_4_ coordination sphere that shows a maximum absorbance at 275 nm (Figure 6). Accordingly, the elution peaks with the lower retention time correspond to the larger Hg(Hk)_2_ complexes featuring the HgS_4_ coordination. In contrast, the peaks at the higher elution volume, indicative of particles of smaller hydrodynamic radius, present negligible 280 nm absorbance and thus can be clearly assigned to the HgHk complexes that show negligible Hg−S LMCT bands at this wavelength range (Figure 6). A clear dimerization followed by a dissociation process is observed for the Hk45 peptide where the apo‐form elutes at the monomer‐specific CV value (dashed line), then it dimerizes up to a Hg^II^‐to‐Hk ratio of 0.9 : 2 and subsequently a rearrangement to the HgHk complex occurs under conditions of Hg^II^ excess. The insignificant uncoupling of observed complex composition from the adjusted Hg^II^: Hk45 ratio is method‐specific and indicates the lack of system homogeneity in experimental conditions. In contrast, the apo‐Hk14 peptide elutes at the dimer‐specific CV value (dotted line) and stays in this range when mixed in a 0.9 : 2 ratio with Hg^II^. Increasing Hg^II^ concentration again lead to the formation of the monomeric HgHk complex as for Hk45 (dashed line). These results suggest that in the applied experimental conditions apo‐Hk14 is not a monomer but rather a dimer formed either electrostatically or due to thiol oxidation (TCEP was not applied in this experiment due to interactions with Hg^II^), as suggested by the visible peak at 280 nm wavelength at 0.76 CV.

### Anisotropy decay analysis

In order to confirm the size‐exclusion results of Rad50 zinc hook assembly in complex with Hg^II^ we analyzed the anisotropy decay of N‐terminally FAM‐labeled Hk14 and Hk45 titrated with Hg^II^. As the anisotropy decay is correlated with the molecule's dynamics and dimensions it is a very precise technique to measure the oligomeric state of proteins and fluorescently labeled peptides.^[53]^ Fitting anisotropy decays provides the rotational correlation time (*τ*_1_)–a parameter that delivers information on molecular dimensions in terms of their diffusion in a solvent. As *τ* describes how fast a fluorophore rotates, thus reducing its anisotropy, it is directly correlated with the hydrodynamic radius of emitting molecules. Rotational correlation time is calculated from Equation (1), as described in the experimental section.

(1) $$\left(ti\right)=r_{0}\sum_{j}g_{j}exp\left(-\frac{t}{\tau_{j}}\right)=\sum_{j}r_{0j}exp\left(-\frac{t}{\tau_{j}}\right)\;$$

where *r*(*t*) is the intensity, $r_{0}=\sum r_{0j}$ is the limiting anisotropy in the absence of rotational diffusion, *τ*_j_ are the individual rotational correlation times and *g*_j_ are the fractional amplitudes of each correlation time in the anisotropy decay ($\sum g_{j}=1.0$ ).

**Figure 8:**
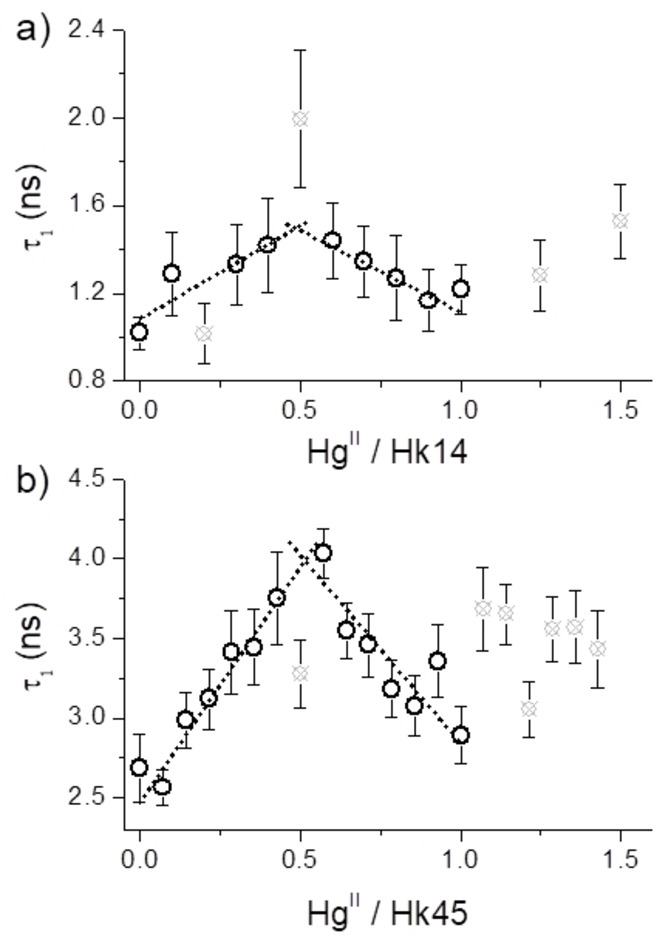
Rotational correlation times (*τ*_1_) of a) Hk14 and b) Hk45 represented as a function of Hg^II^/Hk molar ratio. Error bars represent standard deviation of VV–VH decay difference fitting procedure; gray crossed points were not taken into account during *τ*_1_ vs. Hg^II^/Hk trait fitting. All measurements were carried out in a 50 mM HEPES buffer, pH 7.4, containing 150 mM NaCl and 3.5 μM DTT.

Figure 8 shows rotational correlation times of FAM‐Hk14 and FAM‐Hk45 as a function of the Hg^II^‐to‐Hk molar ratio in the presence of 10× excess of DTT over the peptide. Addition of DTT protects the protein from oxidation, buffers the free Hg^II^ concentration in solution and did not influence the measured parameters unlike other Hg^II^ chelators tested. Both peptides present a linear increase of *τ* from 0 to 0.5 molar equivalents of Hg^II^, which corresponds to the formation of Hg(Hk)_2_ complexes. Afterwards, *τ* decreases to apo‐like values, in line with previous results showing preferential formation of monomeric HgHk complexes at 1.0 molar equivalent of Hg^II^. At the excess of Hg^II^ (>1 molar equivalent) *τ* values are scattered, which suggests the presence of complex equilibria; hence these points (gray crossed circles) were omitted from linear fitting and are not informative regarding the oligomeric state of the analyte. Interestingly, FAM‐Hk45 with a 100‐fold excess of DTT over peptide behaved substantially different and showed only a dimerization‐related *τ* increase and a negligible decrease at higher Hg^II^ molar equivalent values (Figure S5). This experiment suggests that while even a 100‐fold excess of DTT is not Hg^II^‐competitive in the presence of the Hg(Hk45)_2_ complex, it is a stronger competitor than the HgHk45 monocomplex, which simply does not form in such conditions.

### Competition of Hk peptides and low molecular weight thiols for Hg^II^

As pointed out above, estimation of the apparent formation constant of Hg(Hk)_2_ complexes based on anisotropy decay experiments in the presence of DTT is impossible due to the lack of competition. Furthermore, the stability constant(s) of Hg(DTT)_*x*_ complex(es) is not provided in the literature and cannot be used here for stability constant estimation. However, if one assumes that its constant is comparable to BAL,^[54]^ a mercaptan, which like DTT contains two thiol groups, then it can be concluded that the HgHk45 monocomplex presents a slightly higher stability, while Hg(Hk45)_2_ is substantially stronger and approaches covalent‐like forces. We attempted to perform competitivity studies with a series of Hg^II^ chelators, including cyanides and DTT. However, regardless of the experimental conditions applied, Hg(Hk45)_2_ outcompeted the chelator, indicating the absence of metal‐buffering capacity in the desired affinity range. In fact, metal‐mediated folding of the hook domain peptide models applied in this work is completely unaffected by the Hg^II^‐binding competitor (Figure S6), indicating that the protein, due to its high affinity, is able to sequester the desired amount of the metal irrespective of the presence of a competitive ligand.

### ITC study: Considerations of Hg^II^ to Rad50 zinc hook affinity

Spectroscopic investigations on the competition between Hg(Hk)_2_ complexes and commonly used Hg^II^ chelators indicated that Hk14 and Hk45 Rad50 fragments form extremely stable complexes with Hg^II^, with which known and classically used metal ion competitors are unable to compete and therefore obstruct the determination of stability constants. An alternative method that is frequently used to study metal‐peptide complex affinity is isothermal titration calorimetry (ITC). However, it must be underlined that ITC is not suitable for precise determination of tight affinities, and when applied, may yield underestimated formation constants.[^55^, ^56^] As the system's affinity towards the natural metallic cofactor, Zn^II^,^[39]^ exceeds the range of applicability of the method, direct determination of affinity constants from ITC experiments is not possible for metal ions known for their extreme thiophilicity, such as Cd^II^ or Hg^II^.

In our approach, we assumed that the entropy changes (Δ*S*°) caused by Zn^II^, Cd^II^ and Hg^II^ coordination to Hk14 are comparable to each other due to the high structure similarity of the formed complexes and allow to calculate the free energy (Δ*G*) from Equation (2), when Δ*H*° of Zn^II^, Cd^II^ and Hg^II^ complexation is known.

(2) $$\Delta G{}^{\circ }=\Delta H{}^{\circ }-T\times \Delta S{}^{\circ }$$

The enthalpy change for Zn^II^ and Cd^II^ binding to Hk14 in form of the M^II^(Hk14)_2_ complex as well as the stability constant *K*_12_ [Eq. (3)] was determined previously using ITC.[^33^, ^39^] *K*_12_ is the cumulative formation constant of the ML_2_ species, which omits formation of the ML complex for simplicity or necessity in case of high cooperativity.

(3) $$K_{12}=\frac{\left[HgHk_{2}\right]}{\left[Hg^{II}\right]\times {\left[Hk\right]}^{2}}$$

**Figure 9:**
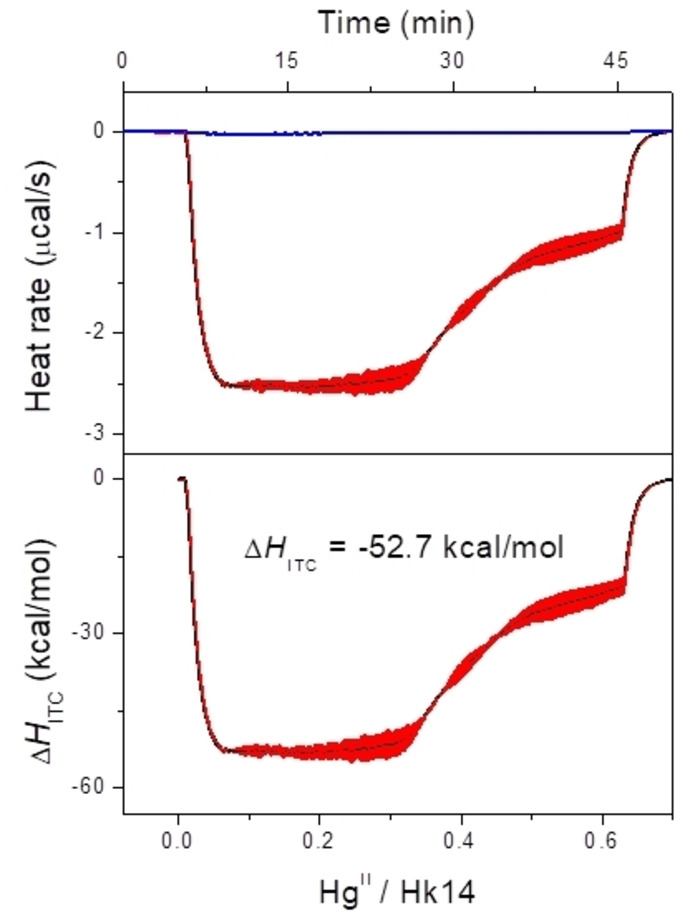
a) ITC analysis of 200 μM Hk14 continuously titrated with 1.3 mM Hg^II^, represented as a baseline‐subtracted heat rate as a function of time. b) Heat converted to molar enthalpy (ΔH_ITC_) as a function of the Hg^II^/Hk14 molar ratio. Presented graphs show the average of two titrations with the standard deviation for each point marked in red. All experiments were performed in 50 mM HEPES buffer, pH 7.4, in a buffer containing 150 mM NaNO_3_.

*K*_12_ was then used to calculate Δ*G* of Zn^II^ and Cd^II^ complexation [Eq. 4]

(4) $$\Delta G{}^{\circ }=-R\times T\times \ln K{}_{12}$$

and subsequently the entropic factors, *T*Δ*S*°, were obtained [Eq. (2), Table 1]. ▵*H*_ITC_ for the Hk14 complexation with Hg^II^ was determined using continuous ITC (cITC) as presented in Figure 9.[^57^, ^58^, ^59^]

**Table 1:** Thermodynamic parameters of Zn^II^, Cd^II^, and Hg^II^ complexation to Hk14. The apparent log *K*_12_ for Hg^II^ complexation at pH 7.4 was calculated assuming a *T*Δ*S*° value identical as for Zn^II^, Cd^II^ or their averaged value.

Parameter	Zn^II[33,39]^	Cd^II[33]^	Hg^II[a]^
Mean Δ*H*_ITC_ [kcal mol^−1^]	−18.1	−21.6	−52.7
*T*Δ*S*° [kcal mol^−1^]	8.08	7.27	n. d.
log*K*_12_ (Hk14)	19.2	21.2	44.2,^[b]^ 44.5,^[c]^ 43.9^[d]^
			44.2 (av.)
log*K*_12_ (Hk45)	20.7	22.6	45.7
log*K*_12_ (Hk130)	20.8	22.7	45.8

[a] This study. [b] Value calculated assuming Δ*S* ^Hg^=(Δ*S* ^Zn^+Δ*S* ^Cd^ )/2. [c] Value calculated assuming Δ*S* ^Hg^=Δ*S* ^Zn^. [d] Value calculated assuming Δ*S* ^Hg=^Δ*S* ^Cd^. n. d.: not determined.

The use of cITC was necessary as we found that the Hg(NO_3_)_2_ concentration in 50 mM HEPES, pH 7.4 (0.15 M NaNO_3_) diminished over time. To reduce the effect of possible loss of Hg^II^ and to minimize the possible peptide oxidation the experimental time had to be kept to the minimum. Δ*H*_ITC_^**=**^−52.7 kcal mol^−1^ was calculated by averaging the plateau corresponding to the formation of a 1 : 2 complex from two independent titrations. Together with the entropic factor, *T*Δ*S*°, of the Zn^II^ and Cd^II^ complexes, the free enthalpy [Eq. (2)] and subsequently the stability constant *K*_12_ for the Hg(Hk14)_2_ complex was calculated [Eq. (4)].[^33^, ^39^] Assuming equal *T*Δ*S*° values for all three M^II^(Hk)_2_ complexes a log Ka12 =44.2 was determined for the Hg(Hk)_2_ complex. Assuming that the entropic factor for the formation of the Hg(Hk)_2_ complex agrees only with the Zn^II^ or the Cd^II^ complex, respectively, slightly different values were obtained (log Kb12 =44.5 vs. log Kc12 =43.9, Table 1). Although these values are calculated based on the equal entropy assumption, they are still experimental values. In addition, as the determination is not based on Hg^II^ competition, it is free from any error of Hg^II^‐competitor stability constants, which could have been under‐ or overestimated in the past. The only constants applied were the *K*_12_ values of Zn(Hk14)_2_ and Cd(Hk14)_2_, which were determined with thoroughly using several independent techniques.[^33^, ^39^] The ITC‐based approach was applied here only for Hk14 due to the system's simplicity; however, one may still estimate log*K*_12_ for Hg(Hk45)_2_ and Hg(Hk130)_2_ based on the known stability constant shift between Zn(Hk14)_2_ and Zn(Hk45)_2_ or between Cd(Hk14)_2_ and Cd(Hk45)_2_. This log*K*_12_ shift is 1.55 for the Zn^II^ and 1.48 for the Cd^II^ hook complexes.[^33^, ^39^] Taking the average value of the shift equal to 1.5, estimated log *K*_12_ for Hg(Hk45)_2_ is 45.7. The analogous log *K*_12_ constant for Hg(Hk130)_2_ based on Cd^II^ complexation by Hk130 is estimated to be 45.8 (Table 1).^[33]^

In order to compare the apparent formation constants of the Hg(Hk)_2_ complexes with Hg^II^ complexes with a 1 : 1 metal‐to‐ligand molar ratio, it is convenient to use the competitivity index (CI), which simplifies various stoichiometries in particular conditions (for details, see Table 2 footnote).[^58^, ^61^] To place the determined stability constants of Hg^II^‐Rad50 zinc hook complexes in a wider spectrum, it is best to compare them with the values evaluated for complexes of ligands representing chemically diverse classes, including classical chelating agents and thiolate compounds (Table 2). For instance, CI values of Hg^II^ complexes of iminodiacetic acid (IDA), nitriloacetic acid (NTA), *N*‐(2‐hydroxyethyl)ethylenediaminetriacetic acid (HEDTA), or ethylenediaminetetraacetic acid (EDTA) increase due to a more pronounced chelate effect and span the range from ∼13 to ∼20. Even in the case of the common potent chelator EDTA, the CI values of Hk complexes are nearly 25 orders of magnitude higher, reaching covalent bond strength. Ligands with established stability constants such as cyanides, cysteine, and N‐acetylcysteine, which have been used in this study, have significantly lower CI values. For that reason they are not able to compete with Hk peptides for Hg^II^. The strongest Hg^II^ ligand described to date, the MerR protein, remains approximately three orders of magnitude weaker compared to the Hk peptides (Table 2). What factors contribute to such high stability of Hg^II^ complexes with hook domain fragments? The most important factor is the enthalpy effect from as many as four highly energetic Hg−S bonds formed during metal complexation. Other Cys‐containing ligands form Hg^II^ sites with digonal or T‐shaped geometry and two or three Hg−S bonds, respectively, at neutral pH.[^26^, ^28^, ^51^] Moreover, Hg^II^ binding causes major structural rearrangements associated with formation of the zinc hook domain in the metal‐coupled folding process. This additionally elevates the stability of the Hg^II^ complex due to the formation of β‐hairpin and energetically favored electrostatic and hydrophobic interactions, which contribute to the free energy of the complexation process.[^33^, ^39^] It is worth emphasizing that symmetrical hook domain formation is possible only in the case of tetrahedral coordination. On the other hand, substantial energy release associated with folding adjustments and general stability increase^[39]^ makes Hg^II^ more prone to form a tetrahedral center, quite uncommon for Hg^II^ complexes with natural products at neutral pH and until now never detected for interprotein assemblies in their natural milieu.

**Table 2:** Affinities of selected low‐molecular‐weight ligands, peptides, and proteins for Hg^II^, forming highly stable complexes, collected across the literature. Apparent formation constant for pH 7.4 was calculated only for ML_2_ complex based on protonation and stability constants determined by specified method in cited reference. Competitivity index was used to simplify stoichiometry of all formed Hg^II^ complexes in order to compare various ligands presented in the table.

Ligand	Method of determination	Conditions	Hg^II^ species stoichiometry^[a]^	Apparent formation constant log*K*_1_, pH 7.4	Apparent formation constant log*K*_12,_ pH 7.4	CI^[b]^	Ref.
l‐Cys	potentiometry	100 mM NaClO_4_, 25 °C	ML, ML_2_	32.3	35.5	33.6	[88]
							[89]
	potentiometry, radiometry	protonation constants: 100 mM NaClO_4_, radiometry: 1 M NaClO_4_	ML_2_	n. c.	33.9	30.6	[90]
	potentiometry	0.225 M NaCl, 0.025 M NaI, 25 °C	ML, ML_2_	25.9	37.5	34.2	[91]
*N*‐acetyl‐l‐Cys (NAC)	potentiometry	protonation constant: 0.16 M NaNO_3_, 25 °C, stability constants: 0.1 M NaClO_4_, 25 °C	ML, ML_2_	33.9	41.5	38.2	[92]
							[89]
Reduced glutathione (GSH)	potentiometry	0.09 M NaCl and 0.01 M NaI	ML, ML_2_	23.7	37.4	34.1	[91]
	potentiometry	0.1 M KCl or KNO_3_, competition with DTPA	ML, ML_2_	17.4	30.6	20.7	[93]
	polarography	1 M KNO_3_	ML_2_	n. c.	41.6	38.3	[94]
Mercaptoacetic acid	potentiometry	0.1 M NaClO_4_ or KNO_3_, 25 °C	ML_2_	n. c.	38.4	35.1	[95]
d‐penicillamine (PSH)	potentiometry	0.5 M NaClO_4_	ML_2,_ ML_3_	n. c.	n. d.	31.9	[96]
	potentiometry	0.1 M NaClO_4_	ML, ML_2_	34.5	37.1	34.9	[54]
	potentiometry	0.1 M KNO_3_	ML	12.5	n. c.	12.5	[97]
	potentiometry	0.15 M KNO_3_	ML, ML_2_	13.7	16.2	13.9	[98]
	potentiometry	0.225 M NaCl, 0.025 M NaI	ML, ML_2_	26.9	37.8	34.5	[91]
	potentiometry	0.09 M NaCl, 0.01 M NaI	ML, ML_2_	13.7	24.9	21.6	[91]
2,3‐Dimercaptopropanol‐1 (BAL)	potentiometry	0.1 M NaClO_4_	ML, ML_2_	11.66	42.6	40.9	[54]
Cyanide (CN^−^)	potentiometry	0.1 M NaNO_3_, 20 °C	ML, ML_2_, ML_3_, ML_4_	n. c.	n. c.	27.8	[88]
Chloride (Cl^−^)	potentiometry	0.1 M NaCl	ML, ML_2_, ML_3_, ML_4_	n. c.	n. c.	10.1	[91]
Iodide (I^−^)	potentiometry	0.1 M NaCl	ML, ML_2_, ML_3_, ML_4_	n. c.	n. c.	21.1	[91]
IDA^[c]^	potentiometry	0.1 M NaClO_4_	ML, ML_2_	8.72	16.39	13.11	[99]
NTA^[c]^	potentiometry	0.5 M NaClO_4_	ML	14.6	n. c.	14.6	[95]
HEDTA^[c]^	potentiometry	0.5 M NaClO_4_	ML	20.5	n. c.	20.5	[95]
EDTA^[c]^	potentiometry	0.5 M NaClO_4_	ML	21.5	n. c.	21.5	[95]
MerR protein	competition	100 mM sodium phosphate, 0.5 M NaCl, pH 7.0, 25 °C, 5 mM L‐cysteine	ML (M_2_L_2_)	39.4	n. c.	39.4	[100]
Hk14	ITC	50 mM HEPES, pH 7.4, 150 mM NaNO_3_	ML_2_	n. c.	44.2	40.9	this study
Hk45	ITC	–	ML_2_	n. c.	45.7	42.4	this study
Hk130	ITC	–	ML_2_	n. c.	45.8	42.5	this study

[a] Protonation of presented complexes was omitted for clarity. [b] CI is the logarithm of the apparent dissociation constant of the HgL complex (Hg^II^ complex of theoretical molecule Z), where [HgZ]=Σ_*ijk*_[Hg_*i*_H_*j*_L_*k*_] at given overall component concentrations. The concentrations of Z in calculations were set at 1 mM and Hg^II^ at 0.25 mM^[60]^. [c] IDA: iminodiacetic acid, NTA: nitriloacetic acid, HEDTA: *N*‐(2‐hydroxyethyl)ethylenediaminetriacetic acid, EDTA: ethylenediaminetetraacetic acid. n. c.: not calculated.

### Biological significance of Hg^II^ coordination to Rad50

Rad50 is one of the most evolutionary conserved proteins in nature, as its homologs have been found in a plethora of organisms belonging to all three domains of life, and even in some viruses.^[62]^ To fulfill its functions, thereby allowing for functioning of the entire MRN complex, Rad50 needs to bind Zn^II^ in an intermolecular manner. Mre11 in the MRN complex is responsible for processing the broken DNA ends, while Rad50 plays a more structural role, acting as a long scaffold that can guide the whole MRN complex.^[63]^ This is only possible if Rad50 dimerizes via the zinc hook, where the zinc intermolecular binding site is formed by the engagement of four thiolates, two from each protein unit, and results in a tetrahedral metal binding site. The Rad50 protein of *P. furiosus*, one of the extremophilic species of Archaea, was the first one to be structurally characterized and for a long time has served as a structural reference for all Rad50 homologs.[^32^, ^47^, ^64^] It is worth noting that the first crystal structure of the Rad50 hook domain was published for a holoprotein that sequestered Hg^II^ (PDB ID: 1 L8D)^[32]^ instead of naturally occurring Zn^II^. The former metal ion was used as a isostructural probe for the latter as it gave better crystal resolution. Although the unique tetrahedral environment of Hg^II^ was formed at the protein interface, it was not the essence of the study and thus was not discussed by the authors. In fact, solid state studies, which provide detailed information on spatial position and orientation of the molecule, may not actually represent the natural biologically relevant status of a molecule in a dynamic cellular environment. For this reason, solution studies were more than necessary to confirm the extraordinary, though so far ignored, findings of Hopfner et al.^[32]^ To elucidate whether or not the crystal structure illustrates the physiological picture of the Rad50 protein interaction with Hg^II^, multi‐technique state‐of‐the‐art studies were performed on well‐designed functional peptidyl models of the protein hook domain in the physiological pH range. Application of mass spectrometry, size‐exclusion chromatography, and life‐time fluorescence spectroscopy identified the presence of Hg^II^‐associated peptide dimers, while ^199^Hg NMR, ^199m^Hg‐PAC, circular dichroism, and electronic spectroscopy confirmed the tetrathiolate HgS_4_ environment around the metal ion at the ratio favoring the formation of biscomplex species (M/L≤1 : 2) and digonal HgS_2_ coordination at equimolar ratios. Notably, the thermodynamic stability of the Hg(Cys)_4_ core formed at the protein interface was higher than that of the digonal HgS_2_ complex, as it could effectively compete with DTT used to buffer the Hg^II^ concentration. Application of the state‐of‐the‐art methodology of ITC allowed to evaluate the incredibly high affinity constant of the HgS_4_ species that is six orders of magnitude higher than the one of the strongest previously evaluated Hg^II^ system. This highlights the unique character of the interaction and to our knowledge makes the Rad50 zinc hook domain the most effective protein‐based Hg^II^‐binding motif in nature. Metal‐coupled folding occurring in the hook domain during complexation makes its Zn^II^, Cd^II^ and Hg^II^ complexes significantly more stable than corresponding metallothioneins.[^33^, ^39^] Even if Rad50 displays outstanding Zn^II^‐binding affinities, this interaction may be effectively disturbed in the presence of Hg^II^ ions. As has been mentioned before, to fulfill its functions the MRN complex must dimerize via Rad50’s zinc hook. Recent reports have indicated that switching between the various topological forms that Rad50 may adopt is crucial for binding to DNA substrates, as well as DNA end processing and repair. It is suggested that signals that induce conformation switching can be generated at two faraway poles of Rad50: at the ATPase globular domain and zinc hook, depending on the species placed 200–500 Å away from the globular domain.[^37^, ^65^, ^66^, ^67^, ^68^] This provides clear evidence that the zinc hook is not only an extraordinarily strong Zn^II^ binding site, but is also involved in delicate MRN complex orchestration. This hypothesis is supported by our previous results suggesting that even small mutations such as point amino acid substitutions could damage DNA repair,[^39^, ^65^] let alone the substitution of Zn^II^ by another metal, altering the complex stability. In consequence, this manifestation of mercury toxicity might effectively upset the function of the Rad50 protein and the cell's ability to detect and repair lethal double‐strand DNA breaks. In fact, the accumulation of mercury inside the cell nucleus and its association with non‐histone proteins of chromatin after mercury exposure in mice, rats, or in isolated cells, was detected years ago.[^69^, ^70^, ^71^, ^72^, ^73^, ^74^, ^75^] Hg^II^ ions were also found to inhibit the DNA binding activity of the Cys_2_His_2_ zinc finger protein transcription factor III A and Sp1 in vitro, although no information on the Hg^II^ coordination was provided.[^76^, ^77^, ^78^] Importantly, the affinity of Hg^II^ towards Rad50 hook models examined in this study was found, not surprisingly, to be significantly higher than the affinity towards Zn^II^. Taken together, the zinc hook domain could serve as an excellent target for Hg^II^ binding and the resulting genotoxic activity.

## Conclusions

Our comprehensive solution study reveals that the Rad50 zinc hook domain of *P. furiosus* sequesters Hg^II^ at the protein interface, forming an extremely stable distorted tetrahedral Hg(Cys)_4_ core with a HgS_4_ coordination environment in the physiological pH range. To the best of our knowledge, it is the first example of an interprotein‐based system that preferentially forms pseudotetrahedral and not the digonal Hg^II^ species at neutral pH in solution. Due to the extremely high affinity of Hg^II^ towards the Cys thiolates of the hook, it can easily displace Zn^II^ from its coordination site. This feature makes it potentially genotoxic, as when entering the cell nucleus, Hg^II^ may bind to the Rad50 hook apex and exert a tremendous effect on the functioning of the entire MR(N/X) complex, in which the zinc hook and DNA‐binding globular domain are structurally and functionally intertwined.

## Experimental Section

**Materials**: *N*,*N*‐Diisopropylethylamine (DIEA), 9‐fluorenylmethoxycarbonyl (Fmoc)‐protected amino acids (Fmoc‐Ala‐OH×H_2_O, Fmoc‐Arg(Pbf)‐OH, Fmoc‐Asn(Trt)‐OH, Fmoc‐Asp(O*t*Bu)‐OH, Fmoc‐Cys(Trt)‐OH, Fmoc‐Gln(Trt)‐OH, Fmoc‐Gln(Trt)‐OH, Fmoc‐Glu(O*t*Bu)‐OH, Fmoc‐Gly‐OH, Fmoc‐His(Trt)‐OH, Fmoc‐Ile‐OH, Fmoc‐Leu‐OH, Fmoc‐Lys(Boc)‐OH, Fmoc‐Met‐OH, Fmoc‐Phe‐OH, Fmoc‐Pro‐OH, Fmoc‐Ser(*t*Bu)‐OH, Fmoc‐Thr(*t*Bu)‐OH, Fmoc‐Tyr(*t*Bu)‐OH, Fmoc‐Val‐OH), piperidine, and *O*‐(benzotriazol‐1‐yl)‐*N*,*N*,*N’*,*N’*‐tetramethyluronium hexafluorophosphate (HBTU) were purchased from Iris Biotech GmbH. HEPES and dithiothreitol (DTT) were purchased from Carl Roth GmbH, CdSO_4_ **⋅** 8/3 H_2_O from Fluka Honeywell while NaNO_3_, ZnSO_4_ **⋅**7 H_2_O, Hg(NO_3_)_2_ **⋅**H_2_O were from Sigma–Aldrich. Trifluoroacetic acid (TFA), ethane‐1,2‐dithiol (EDT), thioanisole, anisole, triisopropylsilane (TIPS), guanidine hydrochloride (GdnHCl), 4‐mercaptophenylacetic acid, tris(2‐carboxyethyl)phosphine hydrochloride (TCEP), ethylenediaminetetraacetic acid (EDTA), and HCl (trace metal grade) were from Merck. Diethyl ether, acetic anhydride, dichloromethane (DCM), HgCl_2_ and NaCl were purchased from Avantor Performance Materials Poland S.A. Chelex 100 resin was from Bio‐Rad, 5,5′‐dithiobis(2‐nitrobenzoic acid) (DTNB) was from TCI Europe N.V., TentaGel R RAM and TentaGel S‐NH_2_ resins were from Rapp Polymere GmbH, and dimethylformamide (DMF) and acetonitrile (MeCN) were from VWR. ^199^HgO was purchased from Cambridge Isotope Labs. All of the experiments were performed in chelexed buffers and solutions. All buffers were prepared with Milli‐Q water obtained with a deionizing water system (Merck KGaA).

**Peptide synthesis**: Zinc hook peptides (Hk14 and Hk45) were synthesized by solid‐phase peptide synthesis (SPPS) using an Fmoc‐strategy on a TentaGel RRAM Amide Rink (Rapp Polymere, Tübingen, Germany) resin (substitution 0.2 mmol g^−1^) and a Liberty 1 microwave‐assisted synthesizer (CEM) as described previously.^[79]^ Peptides were N‐terminally acetylated with acetic anhydride or fluorescently modified with a 5(6)‐carboxyfluorescein (FAM) derivative.^[40]^ Cleaved peptides were precipitated and washed with cold diethyl ether and purified on a C_18_ column (Phenomenex) with a gradient of acetonitrile and 0.1 % TFA using a Dionex Ultimate 3000 HPLC system. The identity of peptides was confirmed with an API 2000 Applied Biosystems ESI‐MS instrument. Concentration of thiols was determined using a sulfhydryl‐group reactant, DTNB (*ϵ*_412_ **=**14 150 M^−1^ cm^−1^), prior to each experiment.^[80]^

**Expression and purification of metal‐free Hk130**: The production of PF Hk130 relied on a previously established protocol using the pTYB21 expression vector (IMPACT Protein Purification System, NEB) and *Escherichia coli* BL21‐CodonPlus (DE3)‐RIL strain.^[81]^ Transformed cells were cultivated in 4 L of LB or TB medium, respectively, and grown at 37 °C until OD_600_ was 0.4–0.5, and then induced with 0.1 mM IPTG. Cultures were incubated overnight at 20 °C with vigorous shaking and subsequently collected by centrifugation at 4500 *g* for 20 min at 4 °C. The pellets were resuspended in ice‐cold buffer A (20 mM HEPES, pH 8.0, 500 mM NaCl, 1 mM PMSF, 1 mM TCEP) and lysed by sonication on ice for 30 min, followed by centrifugation at 20 000 *g* for 15 min. Clarified cell extracts were incubated overnight with chitin resin at 4 °C with mild shaking. After the incubation, the resin was washed with 20 bed volumes of buffer A with increased salt concentration (1 M NaCl) to reduce nonspecific binding of other *E. coli* proteins. To induce the cleavage reaction, 25 mL of buffer B (20 mm HEPES, pH 8.0, 500 mM NaCl, 100 mM DTT) was added to the resin, and the mixture was incubated for 36–48 h at room temperature with mild shaking. Eluted protein solutions were acidified to pH ∼2.5–3.0 with 7 % HCl and concentrated to a small volume using AmiconUltra‐4 Centrifugal Filter Units with NMWL of 3 kDa (Merck Millipore, USA). Hk130 protein was purified by reversed‐phase HPLC in a 0.1 % TFA/acetonitrile gradient (Dionex) followed by lyophilization. The identity of the metal‐free Hk130 protein was confirmed by ESI‐MS, using an API 2000 instrument (Applied Biosystems, USA); the average molecular weight (MW) calculated was 15 217.8/15 217.6 Da (calculated/experimental).

**Spectroscopic studies**: The binding properties of the hook peptide were examined using electronic absorption spectroscopy and circular dichroism (CD). The electronic spectra and CD spectra of Hg^II^ complexes of hook peptides were recorded on a Jasco V‐630 spectrophotometer and a Jasco J‐1500 CD spectropolarimeter, respectively, in a 1 mm quartz cuvette in a wavelength range of 200–320 nm to observe LMCT transitions.[^82^, ^83^, ^84^] The experiments were performed in chelexed 20 mM TRIS buffer, pH 7.4, with 0.1 M NaClO_4_ and 150 μM TCEP. 100 μM solution of Hk14 and 50 μM Hk45 fragments were titrated with small aliquots of 50 mM HgCl_2_ to achieve a 0 to 2 molar ratio over the peptide. The application of TCEP as a thiol protective agent was limited as the Hg^II^ precipitates TCEP at higher concentrations. Based on the obtained absorption and ellipticity maxima, the M/L molar ratio was plotted at different wavelength values.

**Mass spectrometry**: High‐resolution mass spectra were obtained on a Bruker Q‐FT MS spectrometer equipped with an Apollo II electrospray ionization source with an ion funnel. The mass spectrometer was operated in the positive ion mode with the following parameters: scan range *m*/*z* 100–4000, dry gas‐nitrogen, temperature 170 °C, ion energy 5 eV. Capillary voltage was optimized to 4500 V to obtain the highest S/N ratio. The small changes of voltage (±500 V) did not significantly affect the optimized spectra. The samples (metal/ligand in a 1 : 2 stoichiometry, ligand**=**1×10^−4^ M) were prepared in a 1 : 1 mixture of 40 mM carbonate buffer and methanol. Variation of the solvent composition down to 5 % of MeOH did not change the speciation. The sample was infused at a flow rate of 3 mL min^−1^. The instrument was calibrated externally with the Tunemix mixture (Bruker Daltonik) in quadratic regression mode. Data were processed using the Bruker Compass Data Analysis 4.0 program. The mass accuracy for the calibration was higher than 5 ppm, enabling together with the true isotopic pattern (SigmaFit) unambiguous confirmation of the elemental composition of the obtained complex.

^**199**^ **Hg NMR**: The three Rad50 fragments, Hk14, Hk45, and Hk130, were dissolved in 10 % D_2_O/90 % H_2_O to obtain 2–3 mM solutions. After Ellman's test assay an appropriate amount of ^199^Hg(NO_3_)_2_ was added to the peptide samples to reach the desired Hg^II^‐to‐Hk peptide ratio and a small amount of concentrated NaOH was used to adjust the pH to 7.4. At this step Hk130 irreversibly precipitated from the solution, while the complexes with Hk14 and Hk45 showed no sign of precipitation. All spectra were collected using a Bruker Avance 500 MHz spectrometer, equipped with a 5 mm BB inverse probe with an actively shielded *z*‐gradient coil tuned to 89.53 MHz for ^199^Hg. A solution of HgCl_2_ in D_2_O was used as an external reference, setting its peak at a position at −1560 ppm relative to Hg(CH_3_)_2_ (*δ*=0 ppm).^[85]^ The spectral width of 1675 ppm was sampled using 30 000—80 000 scans. The spectra were processed using the software TopSpin (version 4.05).

^**199m**^ **Hg perturbed angular correlation (PAC) spectroscopy**: PAC experiments provide information on the local structure and nanosecond dynamics at the probe site.^[86]^ The experiments were conducted at ISOLDE/CERN in 2018, and the production of the radioisotope ^199m^Hg was carried out as described by Iranzo et al.^[46]^ The protein was prepared by resuspending lyophilized peptides in TRIS buffer (100 mM, pH 7.5 and 8.5). The following stock solutions were prepared and used for the PAC experiments: PF Hk14 (3.3 mM, concentration determined by Ellman's test),^[80]^ Hk45 (1.8 mM), Hk130 (1.5 mM), TRIS buffer (1 M, pH 8.5), and HgCl_2_ (2.0 mM). The final samples contained the appropriate buffer (100 mM), peptide (200 μM or 100 μM), HgCl_2_ (100 μM) and sucrose (55 % *w*/*w*). The sucrose was added to slow the tumbling of proteins due to Brownian motion. A digital PAC instrument (DigiPAC) was used for these experiments, with a time per channel of 0.04883 ns, and a time resolution of 0.7 ns. The data collection and analysis were done with Prelude and Winfit software (developed by Tilman Butz et al., personal communication). 600 data points were used in the fits, excluding the first 9 points due to systematic errors in these. Fourier transformation was carried out using the same 600 data points and a Kaiser‐Bessel parameter of 10. Each nuclear quadrupole interaction (NQI) was modeled using a different set of parameters (*ν*_Q_, *η*, *δ*, and *A*) in addition to a parameter representing the rotational correlation time common to both NQIs, *τ*_c_ (the two NQIs may have different rotational correlation times, but the software does not allow for such analysis). *ν*_Q_ is the quadrupole coupling constant, *η* represents the asymmetry of the electric field gradient at the probe site (attaining a value of 0 in axially symmetric systems, and values up to 1 in a highly asymmetrical system), *δ* is the relative frequency spread, and A is the amplitude of the signal.

**Size‐exclusion chromatography (SEC)**: Gel filtration analyses of P. furiosus Rad50 Hk14 and Hk45 peptides were conducted in 20 mM Tris pH 7.4 with 150 mM NaF using the Dionex Ultimate 3000 HPLC chromatography system equipped with a Shodex KW402.5‐4F column. 5 μL of 1 mM and 0.5 mM Hk14 and Hk45, respectively, dissolved in the running phase, were injected by the autosampler onto the column running at a 0.25 mL min^−1^ flow rate with detector wavelengths set to 220 and 280 nm. Samples were prepared and resolved in the order of 0 to 0.45 and 0.9 molar equivalents of Hg^II^, with a 10‐min incubation period prior the injection and 2 CV column washes between the runs.

**Fluorescence anisotropy decay**: Anisotropy decay studies were performed with a DeltaFlex TCSPC Fluorimeter (Horiba) equipped with a Peltier‐thermostatted cell holder. All measurements were carried out at 294 K in 50 mM HEPES, pH 7.4, 150 mM NaCl, 3.5 μM DTT buffer. 400 μL of 350 nM FAM‐labeled Hk14 and Hk45 was placed in a 1 mL all‐transparent quartz cuvette and excited with a linearly polarized laser from DeltaDiode DD‐485L (wavelength of 485±10 nm, operating at 8.4 MHz) through a 4 nm slit. Emission data were detected at 521 nm wavelength with a sequentially changing polarizer from 0° to 90° until accumulation of a 10,000 peak difference at a 100 ns timescale was reached. Time of a single measurement was set to 1 s. Decay data were subsequently fitted with DAS6 software using two‐exponential fitting, as VV+VH sum and VV−VH difference, and anisotropy. Given the very small targets (Hk14–Hk45 peptides), only difference spectra from reconvolution anisotropy analyses were taken into account.^[33]^

**Isothermal titration calorimetry (ITC)**: The binding of Hg^II^ to Hk14 was monitored using a Nano‐ITC calorimeter (TA Instruments, USA) at 25 °C with an active cell volume of 1 mL. All experiments were performed in 50 mM HEPES buffer, pH 7.4, containing 150 mM NaNO_3_. The use of NaNO_3_ in case of titration with Hg^II^ was deliberate, and was dictated by the fact of the interaction of Hg^II^ and Cl^−^. The Hk14 concentration was 0.2 mM, whereas that of the titrant (ZnSO_4_, CdSO_4_ or Hg(NO_3_)_2_) was 1.3 mM. All titrant solutions were prepared freshly just before measurement, and concentration were confirmed with PAR assays.^[87]^ After initial temperature equilibration, a total volume of 98.01 μL of the titrant was injected continuously over 40 min with stirring at 200 rpm. We decided to use continuous ITC (cITC) due to the observable decrease of the Hg(NO_3_)_2_ concentration in the solution. To determine the heats of titrant dilution, control experiments were performed with identical parameters but in the absence of peptide in the cell. The titration data were analyzed in NanoAnalyze (version 3.3.0). First, data were preprocessed in NanoAnalyze by determining the baseline and then subtracting the heats of dilution. From such pre‐processed data ▵*H*_ITC_ was calculated by averaging the plateau corresponding to the formation of the 1 : 2 complex. Data were not fitted to any kind of model, as Hk14 complexes with metal are characterized by a binding constant outside the measurable range.

## Conflict of interest

The authors declare no conflict of interest.

1

## Supporting information

As a service to our authors and readers, this journal provides supporting information supplied by the authors. Such materials are peer reviewed and may be re‐organized for online delivery, but are not copy‐edited or typeset. Technical support issues arising from supporting information (other than missing files) should be addressed to the authors.

Supporting Information

## Data Availability

The data that support the findings of this study are available from the corresponding author upon reasonable request.

## References

[1] D. C. Bebout in Encyclopedia of Inorganic and Bioinorganic Chemistry, “Mercury: Inorganic & Coordination Chemistry”, Wiley, 2011.

[2] M. Enescu , A. Manceau , Theor. Chem. Acc. 2014, 133: 1457.

[3] A. Manceau , K. L. Nagy , Dalton Trans. 2008, 1421–1425. 18322620 10.1039/b718372k

[4] A. G. Orpen , L. Brammer , F. H. Allen , O. Kennard , D. G. Watson , R. Taylor , J. Chem. Soc. Dalton Trans. 1989, S1–S83.

[5] T. L. Cottrell , in The Strengths of Chemical Bonds, Butterworths, London, 1954, p. 310.

[6] N. Govindaswamy , J. Moy , M. Millar , S. A. Koch , Inorg. Chem. 1992, 31, 5343–5344.

[7] S. Chakraborty , D. S. Touw , A. F. Peacock , J. Stuckey , V. L. Pecoraro , J. Am. Chem. Soc. 2010, 132, 13240–13250. 20825181 10.1021/ja101812c PMC3004433

[8] R. R. Crichton , Biological Inorganic Chemistry: A New Introduction to Molecular Structure and Function, 2 ^nd^ ed., Elsevier, Oxford, 2012, p. 460.

[9] R. A. Bernhoft , J. Environ. Public Health 2012, 2012, 460508. 22235210 10.1155/2012/460508 PMC3253456

[10] T. Barkay , S. M. Miller , A. O. Summers , FEMS Microbiol. Rev. 2003, 27, 355–384. 12829275 10.1016/S0168-6445(03)00046-9

[11] W. Bernhard , M. Good , M. Vašák , J. H. Kägi , Inorg. Chim. Acta 1983, 79, 154–155.

[12] A. Krężel , W. Maret , Chem. Rev. 2021, 121, 14594–14648. 34652893 10.1021/acs.chemrev.1c00371

[13] F. Jalilehvand , K. Parmar , S. Zielke , Metallomics 2013, 5, 1368–1376. 23986393 10.1039/c3mt00173c

[14] F. Jalilehvand , B. O. Leung , M. Izadifard , E. Damian , Inorg. Chem. 2006, 45, 66–73. 16390041 10.1021/ic0508932

[15] P. Faller , B. Ctortecka , W. Tröger , T. Butz , M. Vasák , J. Biol. Inorg. Chem. 2000, 5, 393–401. 10907750 10.1007/pl00010668

[16] J. P. Bourdineaud , M. Gonzalez-Rey , M. Rovezzi , P. Glatzel , K. L. Nagy , A. Manceau , Environ. Sci. Technol. 2019, 53, 4880–4891. 30719924 10.1021/acs.est.8b06579

[17] A. Manceau , P. Bustamante , A. Haouz , J. P. Bourdineaud , M. Gonzalez-Rey , C. Lemouchi , I. Gautier-Luneau , V. Geertsen , E. Barruet , M. Rovezzi , P. Glatzel , S. Pin , Chem. Eur. J. 2019, 25, 997–1009. 30426580 10.1002/chem.201804209 PMC6582439

[18] Ò. Palacios , M. Capdevila in Encyclopedia of Metalloproteins (Eds.: R. H. Kretsinger , V. N. Uversky , E. A. Permyakov ), Springer, New York, 2013, pp. 1386–1390.

[19] W. B. Church , J. M. Guss , J. J. Potter , H. C. Freeman , J. Biol. Chem. 1986, 261, 234–237. 3941073 10.2210/pdb3pcy/pdb

[20] L. M. Utschig , J. W. Bryson , T. V. O′Halloran , Science 1995, 268, 380–385. 7716541 10.1126/science.7716541

[21] A. Stratton , M. Ericksen , T. V. Harris , N. Symmonds , T. P. Silverstein , Protein Sci. 2017, 26, 292–305. 27859834 10.1002/pro.3082 PMC5275735

[22] J. A. Domínguez-Calva , M. L. Pérez-Vázquez , E. Serebryany , J. A. King , L. Quintanar , J. Biol. Inorg. Chem. 2018, 23, 1105–1118. 30167892 10.1007/s00775-018-1607-z

[23] M. Łuczkowski , R. De Ricco , M. Stachura , S. Potocki , L. Hemmingsen , D. Valensin , Metallomics 2015, 7, 478–490. 25633876 10.1039/c4mt00274a

[24] J. B. Tran , A. Krężel , J. Proteome Res. 2021, 20,1889–1901. 33502860 10.1021/acs.jproteome.0c00906 PMC8023803

[25] C. Wallin , J. Jarvet , H. Biverstål , S. Wärmländer , J. Danielsson , A. Gräslund , A. Abelein , J. Biol. Chem. 2020, 295, 7224–7234. 32241918 10.1074/jbc.RA120.012738 PMC7247290

[26] V. L. Pecoraro , A. F. A. Peacock , O. Iranzo , M. Łuczkowski , ACS Symp. Ser. 2009, 1012, 183–197.

[27] A. K. Wernimont , D. L. Huffman , A. L. Lamb , T. V. O′Halloran , A. C. Rosenzweig , Nat. Struct. Biol. 2000, 7, 766–771. 10966647 10.1038/78999

[28] M. Łuczkowski , B. A. Zeider , A. V. Hinz , M. Stachura , S. Chakraborty , L. Hemmingsen , D. L. Huffman , V. L. Pecoraro , Chem. Eur. J. 2013, 19, 9042–9049. 23677531 10.1002/chem.201204184 PMC3814132

[29] T. Kochańczyk , A. Drozd , A. Krężel , Metallomics 2015, 7, 244–257. 25255078 10.1039/c4mt00094c

[30] A. Kocyła , J. B. Tran , A. Krężel , Trends Biochem. Sci. 2021, 46, 64–79. 32958327 10.1016/j.tibs.2020.08.011

[31] G. J. Sharples , D. R. Leach , Mol. Microbiol. 1995, 17, 1215–1217. 8594339 10.1111/j.1365-2958.1995.mmi_17061215_1.x

[32] K. P. Hopfner , L. Craig , G. Moncalian , R. A. Zinkel , T. Usui , B. A. Owen , A. Karcher , B. Henderson , J. L. Bodmer , C. T. McMurray , J. P. Carney , J. H. Petrini , J. A. Tainer , Nature 2002, 418, 562–566. 12152085 10.1038/nature00922

[33] M. Padjasek , M. Maciejczyk , M. Nowakowski , O. Kerber , M. Pyrka , W. Koźmiński , A. Krężel , Chem. Eur. J. 2020, 26, 3297–3313. 31846102 10.1002/chem.201904942 PMC7155053

[34] A. Syed , J. A. Tainer , Annu. Rev. Biochem. 2018, 87, 263–294. 29709199 10.1146/annurev-biochem-062917-012415 PMC6076887

[35] E. Casari , C. Rinaldi , A. Marsella , M. Gnugnoli , C. V. Colombo , D. Bonetti , M. P. Longhese , Front. Mol. Biosci. 2019, 6, 43. 31231660 10.3389/fmolb.2019.00043 PMC6567933

[36] T. H. Stracker , J. H. Petrini , Nat. Rev. Mol. Cell Biol. 2011, 12, 90–103. 21252998 10.1038/nrm3047 PMC3905242

[37] J. Lafrance-Vanasse , G. J. Williams , J. A. Tainer , Prog. Biophys. Mol. Biol. 2015, 117, 182–193. 25576492 10.1016/j.pbiomolbio.2014.12.004 PMC4417436

[38] T. Kochańczyk , P. Jakimowicz , A. Krężel , Chem. Commun. (Camb.) 2013, 49, 1312–1314. 23303248 10.1039/c2cc38174e

[39] T. Kochańczyk , M. Nowakowski , D. Wojewska , A. Kocyła , A. Ejchart , W. Koźmiński , A. Krężel , Sci. Rep. 2016, 6, 36346. 27808280 10.1038/srep36346 PMC5093744

[40] Y. B. Park , M. Hohl , M. Padjasek , E. Jeong , K. S. Jin , A. Krężel , J. H. Petrini , Y. Cho , Nat. Struct. Mol. Biol. 2017, 24, 248–257. 28134932 10.1038/nsmb.3369 PMC5625350

[41] M. Padjasek , A. Kocyła , K. Kluska , O. Kerber , J. B. Tran , A. Krężel , J. Inorg. Biochem. 2020, 204, 110955. 31841759 10.1016/j.jinorgbio.2019.110955

[42] O. P. Ajsuvakova , A. A. Tinkov , M. Aschner , J. B. T. Rocha , B. Michalke , M. G. Skalnaya , A. V. Skalny , M. Butnariu , M. Dadar , I. Sarac , J. Aaseth , G. Bjørklund , Coord. Chem. Rev. 2020, 417, 213343. 32905350 10.1016/j.ccr.2020.213343 PMC7470069

[43] R. A. Pufahl , C. P. Singer , K. L. Peariso , S. J. Lin , P. J. Schmidt , C. J. Fahrni , V. C. Culotta , J. E. Penner Hahn , T. V. O'Halloran , Science 1997, 278, 853–856. 9346482 10.1126/science.278.5339.853

[44] G. Veglia , F. Porcelli , T. DeSilva , A. Prantner , S. J. Opella , J. Am. Chem. Soc. 2000, 122, 2389–2390.

[45] T. Butz , W. Tröger , T. Pöhlmann , O. Nuyken , Z. Naturforsch. A 1992, 47, 85–88.

[46] O. Iranzo , P. W. Thulstrup , S. B. Ryu , L. Hemmingsen , V. L. Pecoraro , Chem. Eur. J. 2007, 13, 9178–9190. 17960740 10.1002/chem.200701208

[47] A. Jancsó , J. G. Correia , R. K. Balogh , J. Schell , M. L. Jensen , D. Szunyogh , P. W. Thulstrup , L. Hemmingsen , Nucl. Instrum. Methods Phys. Res. Sect. A 2021, 1002, 165154.

[48] K. P. Hopfner , A. Karcher , L. Craig , T. T. Woo , J. P. Carney , J. A. Tainer , Cell 2001, 105, 473–485. 11371344 10.1016/s0092-8674(01)00335-x

[49] J. R. Winther , C. Thorpe , Biochim. Biophys. Acta. 2014, 1840, 838–884. 23567800 10.1016/j.bbagen.2013.03.031 PMC3766385

[50] W. Lu , M. Stillman , J. Am. Chem. Soc. 1993, 115, 3291–3299.

[51] M. Łuczkowski , M. Stachura , V. Schirf , B. Demeler , L. Hemmingsen , V. L. Pecoraro , Inorg. Chem. 2008, 47, 10875–10888. 18959366 10.1021/ic8009817 PMC2650386

[52] T. J. Graddis , D. G. Myszka , I. M. Chaiken , Biochemistry 1993, 32, 12664–12671. 8251485 10.1021/bi00210a015

[53] J. R. Lakowicz , in Principles of Fluorescence Spectroscopy, Springer, Berlin, 2010, p. 954.

[54] J. S. Casas , M. M. Jones , J. Inorg. Nucl. Chem. 1980, 42, 99–102.

[55] K. Kluska , J. Adamczyk , A. Krężel , Coord. Chem. Rev. 2018, 367, 18–64.

[56] A. Miłoch , A. Krężel , Metallomics 2014, 6, 2015–2024. 25109667 10.1039/c4mt00149d

[57] O. Alkhamis , W. Yang , R. Farhana , H. Yu , Y. Xiao , Nucleic Acids Res. 2020, 48, e120. 33053182 10.1093/nar/gkaa849 PMC7672472

[58] P. Dumas , Eur. Biophys. J. 2022, 51, 77–84. 34999938 10.1007/s00249-021-01588-4

[59] N. Markova , D. Hallén , Anal. Biochem. 2004, 331, 77–88. 15245999 10.1016/j.ab.2004.03.022

[60] A. Krężel , J. Wójcik , M. Maciejczyk , W. Bal , Chem. Commun. (Camb.) 2003, 704–705. 12703782 10.1039/b300632h

[61] A. Krężel , W. Maret , Arch. Biochem. Biophys. 2016, 611, 3–19. 27117234 10.1016/j.abb.2016.04.010 PMC5120989

[62] J. C. Connelly , D. R. F. Leach , Trends Biochem. Sci. 2002, 27, 410–418. 12151226 10.1016/s0968-0004(02)02144-8

[63] L. Käshammer , J. H. Saathoff , K. Lammens , F. Gut , J. Bartho , A. Alt , B. Kessler , K. P. Hopfner , Mol. Cell 2019, 76, 382–394.e6. 31492634 10.1016/j.molcel.2019.07.035

[64] K. P. Hopfner , A. Karcher , D. S. Shin , L. Craig , L. M. Arthur , J. P. Carney , J. A. Tainer , Cell 2000, 101, 789–800. 10892749 10.1016/s0092-8674(00)80890-9

[65] M. Hohl , T. Kochańczyk , C. Tous , A. Aguilera , A. Krężel , J. H. Petrini , Mol. Cell 2015, 57, 479–491. 25601756 10.1016/j.molcel.2014.12.018 PMC4527088

[66] M. Hohl , Y. Kwon , S. M. Galván , X. Xue , C. Tous , A. Aguilera , P. Sung , J. H. J. Petrini , Nat. Struct. Mol. Biol. 2011, 18, 1124–1131. 21892167 10.1038/nsmb.2116 PMC3190017

[67] R. A. Deshpande , G. J. Williams , O. Limbo , R. S. Williams , J. Kuhnlein , J. H. Lee , S. Classen , G. Guenther , P. Russell , J. A. Tainer , T. T. Paull , EMBO J. 2014, 33, 482–500. 24493214 10.1002/embj.201386100 PMC3989629

[68] J. H. Lee , M. R. Mand , R. A. Deshpande , E. Kinoshita , S. H. Yang , C. Wyman , T. T. Paull , J. Biol. Chem. 2013, 288, 12840–12851. 23525106 10.1074/jbc.M113.460378 PMC3642328

[69] S. E. Bryan , C. Lambert , K. J. Hardy , S. Simons , Science 1974, 186, 832–833. 4469682 10.1126/science.186.4166.832

[70] S. E. Bryan , A. L. Guy , K. J. Hardy , Biochemistry 1974, 13, 313–319. 4810055 10.1021/bi00699a013

[71] M. Różalski , R. Wierzbicki , Environ. Res. 1979, 20, 465–9. 546652 10.1016/0013-9351(79)90021-5

[72] M. Różalski , E. Kuziemska , R. Wierzbicki , Biochem. Pharmacol. 1981, 30, 2177–2178. 7295333 10.1016/0006-2952(81)90242-2

[73] M. Różalski , R. Wierzbicki , Biochem. Pharmacol. 1983, 32, 2124–2126. 6223640 10.1016/0006-2952(83)90441-0

[74] M. Christensen , S. C. Mogensen , J. Rungby , Arch. Toxicol. 1988, 62, 440–446. 3250374 10.1007/BF00288347

[75] L. Bucio , C. García , V. Souza , E. Hernández , C. González , M. Betancourt , M. C. Gutiérrez-Ruiz , Mutat. Res. 1999, 423, 65–72. 10029678 10.1016/s0027-5107(98)00226-7

[76] J. S. Rodgers , J. R. Hocker , R. J. Hanas , E. C. Nwosu , J. S. Hanas , Biochem. Pharmacol. 2001, 61, 1543–1550. 11377384 10.1016/s0006-2952(01)00629-3

[77] R. Pamphlett , S. Kum Jew , Front. Med. 2019, 6, 168. 10.3389/fmed.2019.00168 PMC6659129 31380381

[78] R. Pamphlett , L. Satgunaseelan , S. Kum Jew , P. A. Doble , D. P. Bishop , PLoS One 2020, 15:e0228226. 32004334 10.1371/journal.pone.0228226 PMC6993973

[79] A. Pomorski , T. Kochańczyk , A. Miłoch , A. Krężel , Anal. Chem. 2013, 85, 11479–11486. 24180305 10.1021/ac402637h

[80] P. Eyer , F. Worek , D. Kiderlen , G. Sinko , A. Stuglin , V. Simeon-Rudolf , E. Reiner , Anal. Biochem. 2003, 312, 224–227. 12531209 10.1016/s0003-2697(02)00506-7

[81] A. Drozd , D. Wojewska , M. D. Peris-Diaz , P. Jakimowicz , A. Krężel , Metallomics 2018, 10, 595–613. 29561927 10.1039/C7MT00332C

[82] M. Vasák , J. H. Kägi , H. A. Hill , Biochemistry 1981, 20, 2852–2856. 7248252 10.1021/bi00513a022

[83] R. K. Balogh , B. Gyurcsik , É. Hunyadi-Gulyás , J. Schell , P. W. Thulstrup , L. Hemmingsen , A. Jancsó , Chem. Eur. J. 2019, 25, 15030–15035. 31365771 10.1002/chem.201902940 PMC6899792

[84] O. Sénèque , P. Rousselot-Pailley , A. Pujol , D. Boturyn , S. Crouzy , O. Proux , A. Manceau , C. Lebrun , P. Delangle , Inorg. Chem. 2018, 57, 2705–2713. 29443519 10.1021/acs.inorgchem.7b03103

[85] G. Klose , F. Volke , G. Peinel , G. Knobloch , Magn. Reson. Chem. 1993, 31, 548–551.

[86] L. Hemmingsen , K. N. Sas , E. Danielsen , Chem. Rev. 2004, 104, 4027–4062. 15352785 10.1021/cr030030v

[87] A. Kocyła , A. Pomorski , A. Krężel , J. Inorg. Biochem. 2015, 152, 82–92. 26364130 10.1016/j.jinorgbio.2015.08.024

[88] G. Anderegg , Helv. Chim. Acta 1957, 40, 1022–1026.

[89] M. A. Basinger , J. S. Casas , M. M. Jones , A. D. Weaver , N. H. Weinstein , J. Inorg. Nucl. Chem. 1981, 43, 1419–1425.

[90] J. Starý , K. Kratzer , J. Radioanal. Nucl. Chem. 1988, 126, 69–75.

[91] P. Cardiano , G. Falcone , C. Foti , S. Sammartano , J. Chem. Eng. Data 2011, 56, 4741–4750.

[92] A. E. Fazary , N. S. Awwad , H. A. Ibrahium , A. A. Shati , M. Y. Alfaifi , Y. H. Ju , ACS Omega 2020, 5, 19598–19605. 32803054 10.1021/acsomega.0c02080 PMC7424727

[93] P. D. Oram , X. Fang , Q. Fernando , P. Letkeman , D. Letkeman , Chem. Res. Toxicol. 1996, 9, 709–712. 8831814 10.1021/tx9501896

[94] W. Stricks , I. M. Kolthoff , J. Am. Chem. Soc. 1953, 75, 5673–5681.

[95] A. E. Martell , R. M. Smith , in Critical Stability Constants, Vol. 5, Springer, Boston, 1982, p. 604.

[96] H. Kõszegi-Szalai , T. L. Paál , Talanta 1999, 48, 393–402. 18967478 10.1016/s0039-9140(98)00258-6

[97] G. R. Lenz , A. E. Martell , Biochemistry 1964, 3, 745–750. 14211608 10.1021/bi00894a001

[98] E. J. Kuchinskas , Y. Rosen , Arch. Biochem. Biophys. 1962, 97, 370–372. 14460165 10.1016/0003-9861(62)90090-5

[99] W. E. Van der Linden , C. Beers , Talanta 1975, 22, 89–92. 18961624 10.1016/0039-9140(75)80148-2

[100] J. Parkhill , A. Z. Ansari , J. G. Wright , N. L. Brown , T. V. O′Halloran , EMBO J. 1993, 12, 413–421. 8440234 10.1002/j.1460-2075.1993.tb05673.x PMC413224

