# Supplementary material for: An Extremely Stable Interprotein Tetrahedral Hg(Cys)4 Core Forms in the Zinc Hook Domain of Rad50 Protein at Physiological pH
Source: Chemistry. 2022 Nov 7;28(66):e202202738. doi: 10.1002/chem.202202738 (PMC9828754; doi:10.1002/chem.202202738)
Supplement: Supplementary file 1 — Supporting Information [file CHEM-28-0-s001.pdf]

Chemistry–A European Journal

Supporting Information

An Extremely Stable Interprotein Tetrahedral $Hg(Cys)_4$ Core Forms in the Zinc Hook Domain of Rad50 Protein at Physiological pH

Marek Łuczkowski, Michał Padjasek, Józef Ba Tran, Lars Hemmingsen, Olga Kerber, Jelena Habjanič, Eva Freisinger, and Artur Krężel*

TABLE OF CONTENTS

Figure S1. Mre11-Rad50 complex architecture……………………………………………S3

Table S1. Calculated and experimental mass values of synthesized and expressed peptides and their sequences………………………………………..S4

Table S2. Two different fits to the sum of all ^{199m}Hg PAC data………………………………S5

Figure S2. Two different fits to the sum of all ^{199m}Hg PAC spectra…………………………...S6

Table S3. Parameters fitted to ^{199m}Hg PAC spectra……………………………….……..…S7

Figure S3. ^{199m}Hg PAC data for the Hk14, Hk45 and Hk130 peptides………….…………S8

Figure S4. Hg^{II} CD spectra – metal free spectrum = coiled coil…………………..………S9

Figure S5. Rotational correlation times (τ_1) of Hk45 represented as a function of Hg^{II}/peptide molar ratio…………………….…...S10

Figure S6. CD spectra of Rad50 zinc hook fragments titrated with Hg^{II}……………….………S11

References…………………………………………………………………………..S11

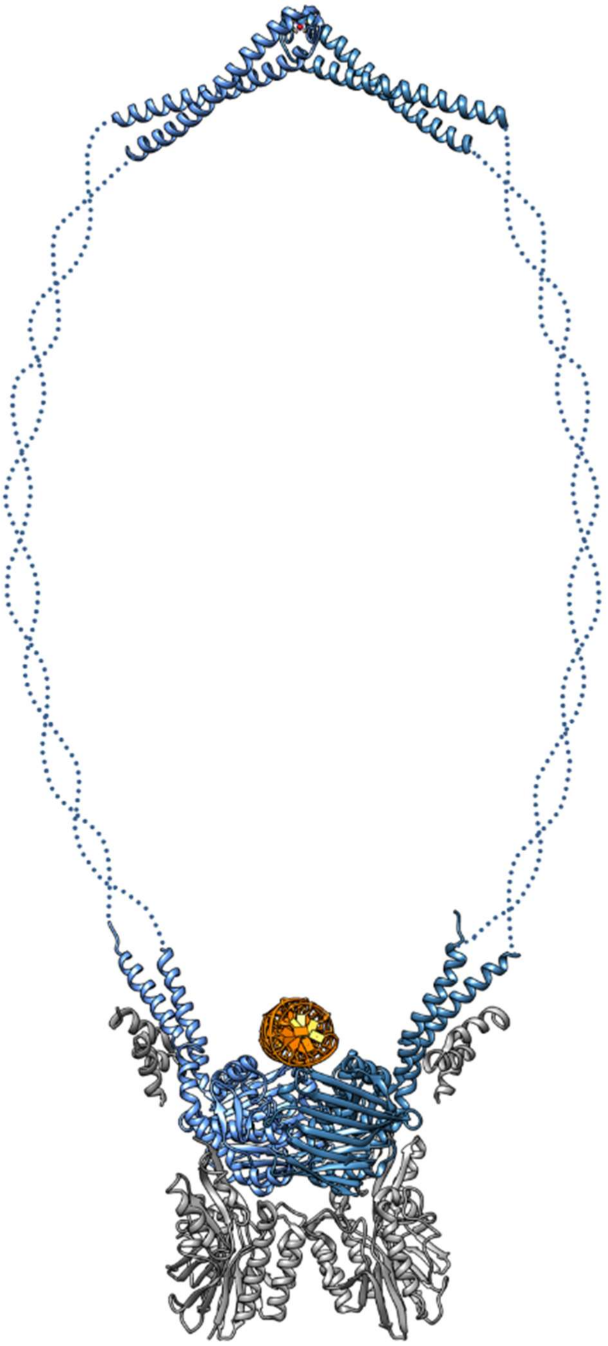

Figure S1. Mre11-Rad50 complex architecture. The scheme represents crystal structures of *P. furiosus* hook complexed with Hg(II) (PDB code: 1L8D)[1] and DNA-bound globular Rad50-Mre11 apex from *Methanocaldococcus jannaschii* (PDB code: 5F3W)[2] connected via long (100-700 Å) coiled-coil domains. Blue, gray and orange represent two Rad50, two Mre11 and one DNA molecule, respectively. Hg(II) is shown as a red sphere.

Table S1. Calculated and experimental mass values of synthesized and expressed peptides and their sequences. *m* and *av* refer to monoisotopic and averaged masses, respectively. Ac, NH_2, and FAM stand for acetyl, amide and fluorescein modification, respectively.

Zinc hook peptide	MW calculated / Da	MW experimental / Da
**Hk14** (N-Ac and C-NH_2)	1 517.8^{av}	1 518.4^{av}
**Hk45** (N-Ac and C-NH_2)	5 164.8^{av}	5 165.8^{av}
**Hk130** (N- and C-termini free)	15 217.8^{av}	15 217.6^{av}
**FAM-Hk14** (N-FAM, C-NH_2)	1834.0^{av}	1835.4^{av}
**FAM-Hk45** (N-FAM, C-NH_2)	5481.1^{av}	5483.2^{av}

Sequence of Hk130 (*P. furiosus*). Hk14 fragment is marked in red, while Hk45 is in blue and red:

RQLKEKLGDKSPEDIKKLLEELETKKTTIEEERNEITQRIGELKN**KIGDLKTAIEELKKAKG**
KCPVCGRELTDEHREELLSKYHLDLNNSKNTLAKLIDRKSELERELRRIDMEIKRLTPLLTV
AEQIRS

Table S2. Two different fits to the sum of all ^{199m}Hg PAC data; see Figure S2. Numbers in parentheses are standard deviations of the fitted parameters; see Figure S2.

Fit	ν_Q (GHz)	η	δ ×100	$1/\tau_c$ μs^{-1}	A ×100	χ_r^2
Rapidly reorienting NQI and high frequency NQI	1.66(1)	0.17(3)	4(1)	0(23)<br>327(16)	3.9(4)<br>11.1(2)	1.01
Low frequency NQI and high frequency NQI (with low η)	1.65(1)<br>0.20(2)	0.13(4)<br>1(1)	6(2)<br>17(14)	52(33)	6.6(7)<br>8(1)	0.76

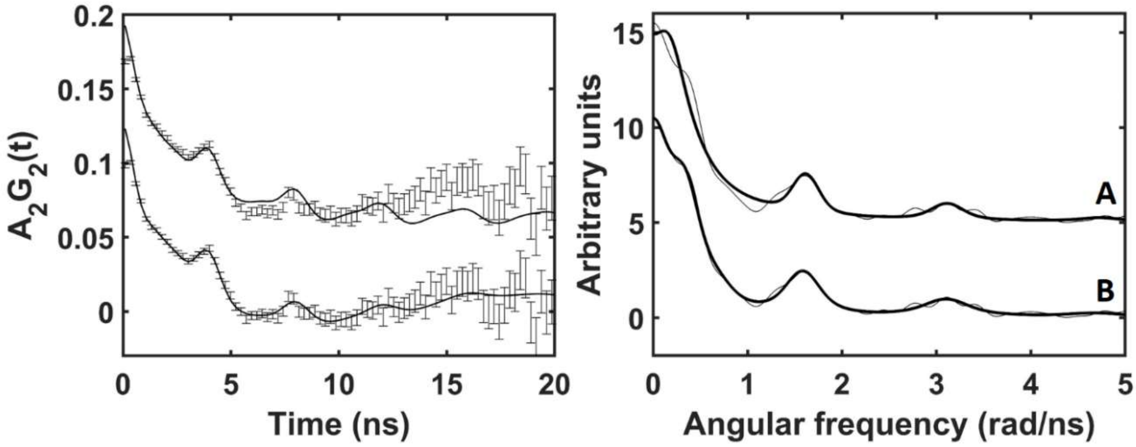

Figure S2. Two different fits to the sum of all ^{199m}Hg PAC spectra; see Table S2 for the fitted parameters. Left panel: perturbation function (data with error bars displayed as averages of 5 points, fit as full line). Right panel: Fourier transformed data (thin line) and fit (bold faced line). The main point of this analysis is to discriminate between two different interpretations of the data: A: the data are analyzed with two components, a high frequency NQI giving rise to rapid oscillations (maxima at t = 0, t ~ 3.5 ns, t ~ 7 ns) and an exponential decay originating from rapid reorientation of the electric field gradient reflecting unspecific binding and rapid exchange dynamics of Hg^{II}; B: the data are analyzed by two (static) NQIs, one high frequency, and the other a very low frequency NQI (maxima at t = 0 and ~16 ns). This analysis demonstrates that scenario A does not fit the data well, as the fit systematically deviates from the data both in the range 5–7 ns and 13–17 ns. Moreover, the reduced chi-square (Table S2) is larger than that for scenario B. This conclusion is important, because it demonstrates that the low frequency signal is indeed present in the data. This low frequency NQI is interpreted as originating from a distorted tetrahedral $\{HgS_4\}$ species.

Table S3. Parameters fitted to ^{199m}Hg PAC spectra. Numbers in parentheses are standard deviations of the fitted parameters. "f" indicates that the parameter was fixed in the fit.

Peptide	Hg(II):Hk	pH	ν_Q	η	δ	$1/\tau_c$	A	χ_r^2
			(GHz)		×100	us^{-1}	×100	
Hk14	1:1	7.4	1.48(1)<br>0.2f	0.22(3)<br>1f	2(2)<br>17f	105(44)	14(1)<br>1.4(5)	0.77
Hk14	1:2	7.4	1.62(3)<br>0.15(2)	0.18(7)<br>1f	8(3)<br>20(32)	35(55)	7(1)<br>8(2)	0.80
Hk14[a]	1:2	8.5	0.10(3)	1f	17f	133(54)	13(1)	0.72
Hk45	1:2	7.4	1.61(3)<br>0.202(6)	0(1)<br>1f	6(3)<br>4(15)	72(39)	6(1)<br>8.8(9)	0.74
Hk45	1:2	8.5	1.67(2)<br>0.19(1)	0.16(5)<br>1f	1(2)<br>7(19)	102(54)	7(1)<br>6.9(8)	0.78
Hk130	1:2	7.4	1.60(4)<br>0.21(2)	0.23(7)<br>1f	5(4)<br>20(34)	54(77)	5(2)<br>9(2)	0.85
Hk130	1:2	8.5	1.66(4)<br>0.22(1)	0.17(7)<br>1f	8(3)<br>17(14)	36(28)	7(1)<br>9(1)	0.77

[a] Including a high frequency NQI in the fit did not improve the fit significantly.

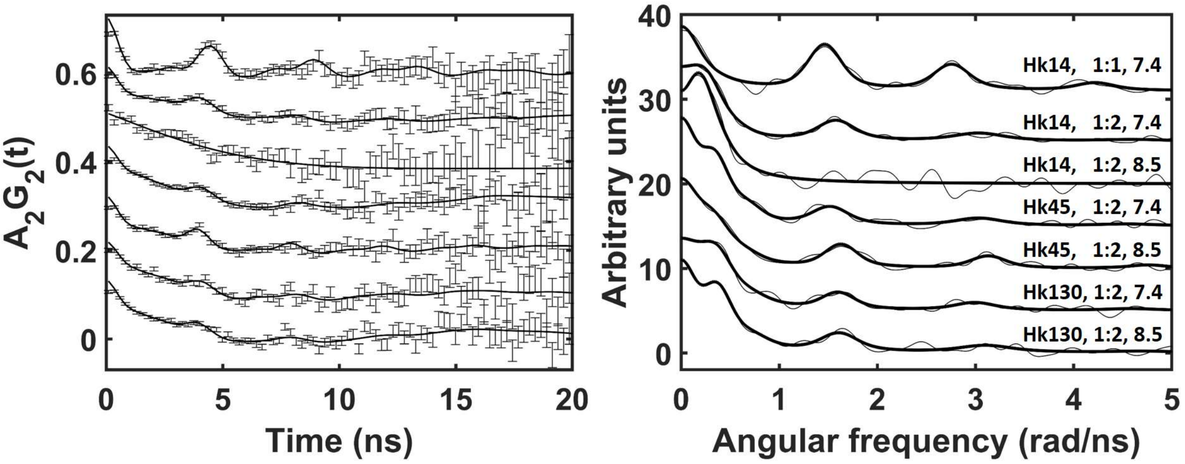

Figure S3. ^{199m}Hg PAC data for the Hk14, Hk45, and Hk130 peptides. Left: Perturbation function (data points with error bars and fit (full line)). Right: Fourier transformed experimental data (thin line) and fit (bold faced line). The indicated conditions pertain to both the left and right panel and refer to peptide, Hg^{II}:Hk, pH.

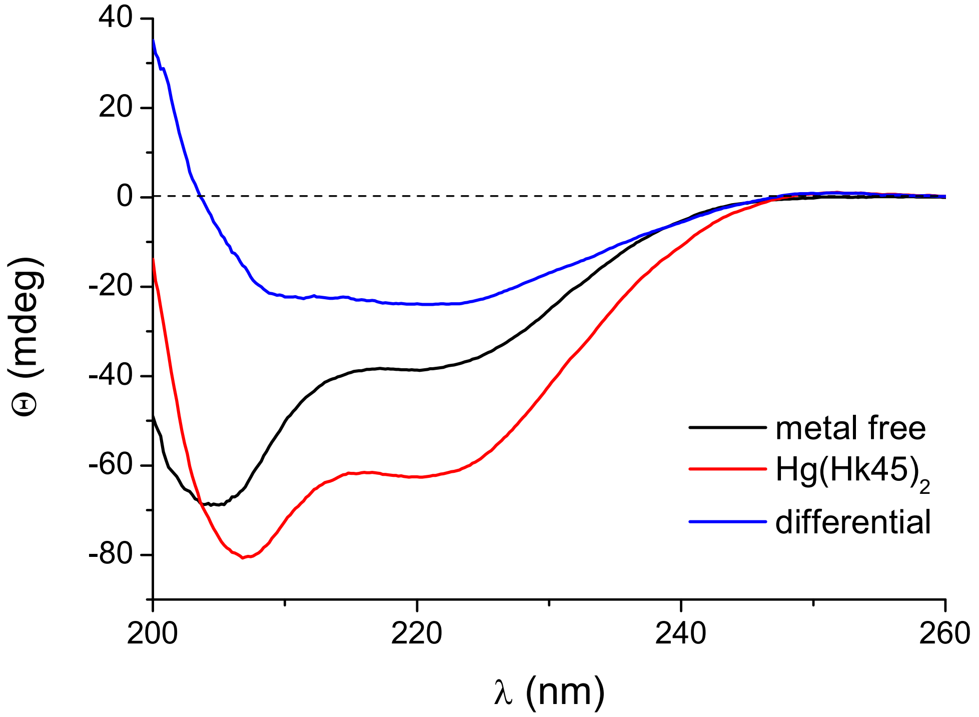

Figure S4. Differential CD spectrum of mercurated and metal-free 50 µM Hk45 model of Rad50 zinc hook domain. Spectra were recorded in 20 mM Tris-HCl buffer, pH 7.4, 0.1 M $NaClO_4$, and 150 µM TCEP. Subtraction yields the canonical spectra of coiled-coil secondary structure.

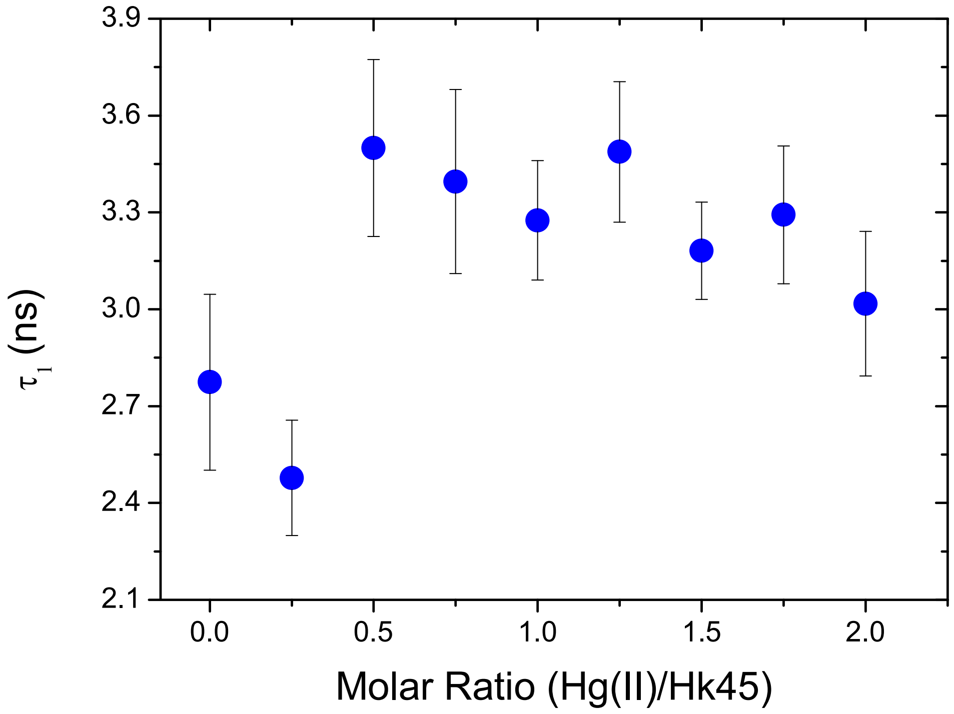

Figure S5. Rotational correlation times (τ_1) of Hk45 represented as a function of Hg^{II}/peptide molar ratio. Experiment conducted with 100× molar excess of DTT over peptide (0.35 μM Hk45 and 35 μM DTT). Error bars represent standard deviation of VV-VH decay difference fitting procedure.

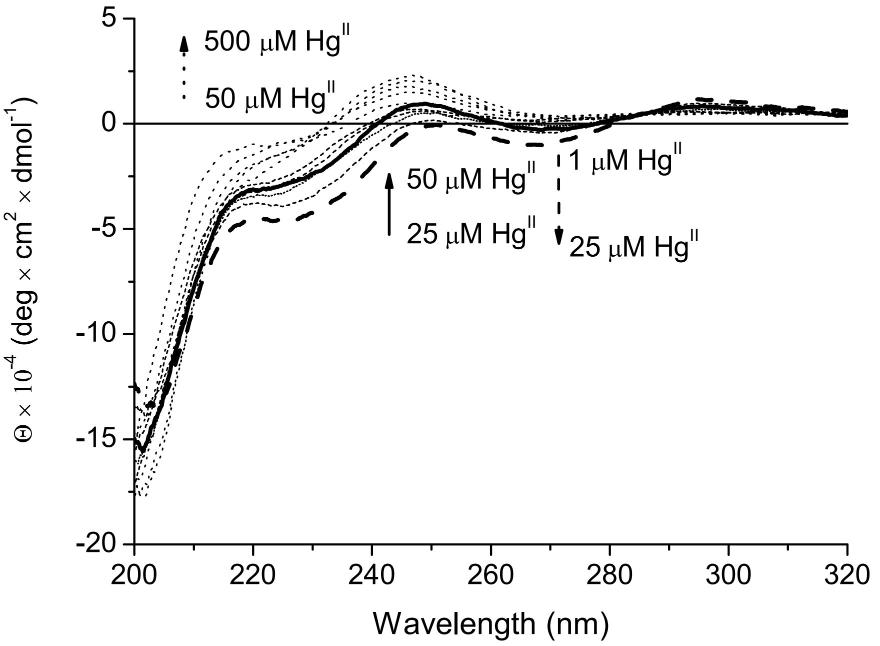
500 μM Hg^II
50 μM Hg^II
50 μM Hg^II
25 μM Hg^II
1 μM Hg^II
25 μM Hg^II
Θ × 10^-4 (deg × cm^2 × dmol^-1)
Wavelength (nm)

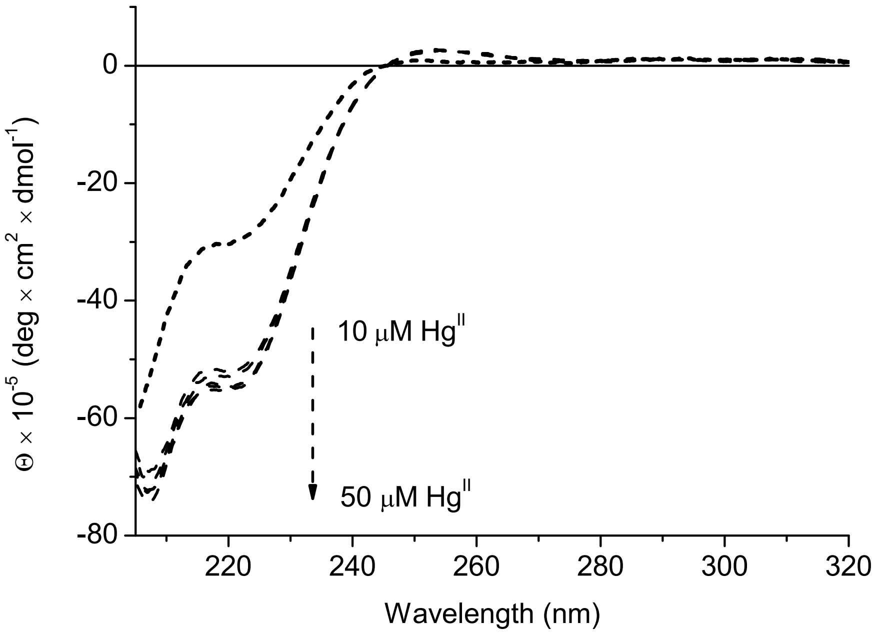
10 μM Hg^II
50 μM Hg^II
Θ × 10^-5 (deg × cm^2 × dmol^-1)
Wavelength (nm)

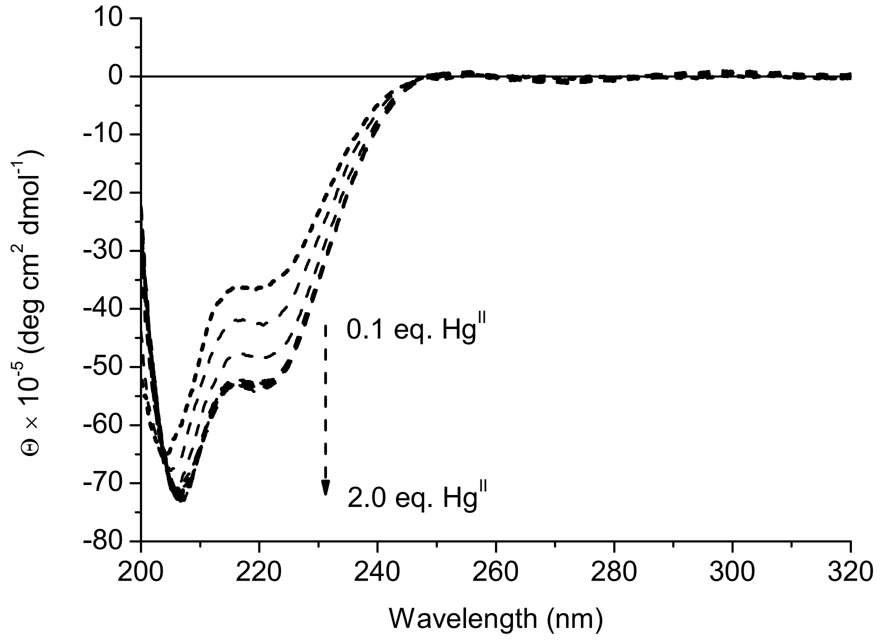

Figure S6. CD spectra of Rad50 zinc hook fragments titrated with Hg^{II}: 50 μM Hk14 in the presence of 5 mM KCN (a), 25 μM Hk45 in the presence of 5 mM KCN (b), and 25 μM Hk45 in the presence of 250 μM DTT (b). Spectra were recorded in 20 mM Tris-HCl buffer pH 7.4, 0.1 M NaF.

References:

[1] K. P. Hopfner, L. Craig, G. Moncalian, R. A. Zinkel, T. Usui, B. A. Owen, A. Karcher, B. Henderson, J. L. Bodmer, C. T. McMurray, J. P. Carney, J. H. Petrini, J. A. Tainer, *Nature* **2002**, *418*, 562–566.

[2] Y. Liu, S. Sung, Y. Kim, F. Li, G. Gwon, A. Jo, A.K. Kim, T. Kim, O.K. Song, S.E. Lee, Y. Cho, *EMBO J.* **2016**, *35*, 743–758.
